# Genetic and Epigenetic Mechanisms Underpinning Biotic Stress Resilience of *Brassica* Vegetables

**DOI:** 10.3390/plants14243765

**Published:** 2025-12-10

**Authors:** Mst. Arjina Akter, Mei Iwamura, Shrawan Singh, Md Asad-Ud Doullah, Ryo Fujimoto, Henrik U. Stotz, Hasan Mehraj

**Affiliations:** 1 Graduate School of Agricultural Science, Kobe University, 1-1 Rokkodai, Nada-ku, Kobe 657-8501, Japan; arjina.ppath@bau.edu.bd (M.A.A.); 250a322a@stu.kobe-u.ac.jp (M.I.); singhshrawan@rediffmail.com (S.S.); 2 Department of Plant Pathology, Faculty of Agriculture, Bangladesh Agricultural University, Mymensingh 2202, Bangladesh; 3 Division of Vegetable Science, ICAR-Indian Agricultural Research Institute, New Delhi 110012, India; 4 Department of Plant Pathology and Seed Science, Faculty of Agriculture, Sylhet Agricultural University, Sylhet 3100, Bangladesh; asad.ppath@sau.ac.bd; 5 School of Health, Medical and Life Sciences, University of Hertfordshire, Hatfield, Hertfordshire AL10 9AB, UK; h.stotz@herts.ac.uk; 6 Department of Applied Biological Chemistry, Graduate School of Agricultural and Life Science, The University of Tokyo, 1-1-1 Yayoi, Bunkyo-ku, Tokyo 113-8657, Japan

**Keywords:** breeding, DNA methylation, histone modification, OMICS, pathogen, quantitative trait loci, resistance

## Abstract

Breeding for disease-resistant varieties is a sustainable solution to reduce substantial production losses caused by pathogenic infestations in *Brassica* vegetables, bypassing environmentally risky disease management practices. Host-resistant genetic mechanisms aid breeders to identify resistance loci and linked markers for the clubroot, Fusarium yellows, downy mildew, black rot, stem rot, soft rot, white rust, and turnip mosaic virus diseases in *Brassica* vegetables. Introgression of the resistance (*R*) genes by marker-assisted selection (MAS) breeding strategies allow the development of disease-resilient varieties. *Brassica rapa* clubroot-resistant genes (*CRa*, *CRc*, *CRd*, *CRk*, and *Crr5*) have been introgressed into Chinese cabbage, while *CR* genes (*CRa*, *CRb*, *CRc*, *Crr1*, *Crr2*, and *Crr3*) from *B. rapa* were also introgressed into *B. oleracea*. Beyond MAS, *R* genes can be precisely engineered by CRISPR-based technologies into precise and durable resistant varieties. The involvement of DNA methylation and histone modifications epigenetically regulate resistance mechanisms, often via ethylene/salicylic acid/jasmonic acid signaling pathways. DNA methylation mediates systemic acquired resistance by the differential expression of genes such as *JAZ1*, *PR3*, and *NDR1*. Future progress will depend on identifying epiQTLs and epi-markers linked to *R* genes. Epigenetic insights with genetic knowledge will facilitate breeding of biotic stress-resilient *Brassica* vegetables. This review synthesizes current molecular understanding of biotic stressors and provides future directions for disease resistance breeding of *Brassica* vegetable plants.

## 1. Introduction

*Brassica* vegetables are major crops of global importance. These vegetables represent various edible organs such as leaves (e.g., cabbage, Chinese cabbage, kale, Brussels sprouts), stems (kohlrabi), and inflorescences (cauliflower, broccoli) used for culinary purposes. *Brassica* vegetables hold the 4th and 13th ranks in production and globally cultivated areas (100.4 million tons from 3.8 million ha [excluding turnip] in 2023, Figure 1 [1]), respectively, when compared to other vegetables. *Brassica oleracea* (cabbage, cauliflower, broccoli, kohlrabi, and kale) and *Brassica rapa* (Chinese cabbage, komatsuna, pak choi, and turnip) are predominantly grown in temperate, subtropical, and tropical regions worldwide. Although *Brassica* vegetables have a high yield potential, their average global productivity remains low because of their susceptibility to both biotic and abiotic stresses. These stressors also affect the quality of *Brassica* vegetables. The resilience of *Brassica* vegetables to biotic stress is one of the prime interests of breeders impacting global food security. Global climate change challenges the resistance mechanisms of existing resistant *Brassica* vegetable varieties, reducing their durability against biotic stresses and hindering resilient production.

**Figure 2:**
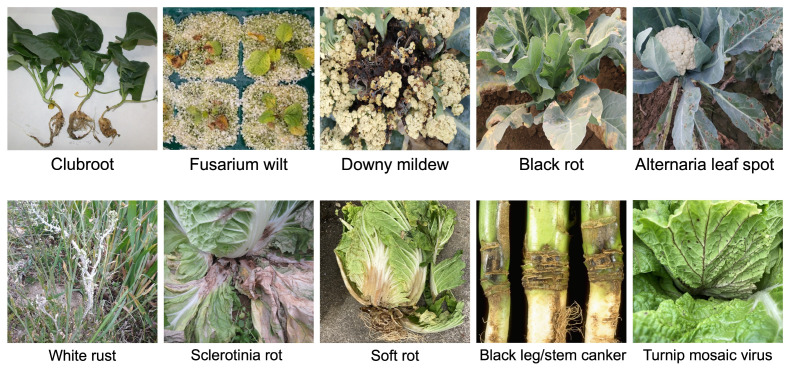
Disease symptoms in *Brassica* genus. A picture of Fusarium wilt/yellows was taken at the seedling stage. White rust and blackleg disease symptoms were taken from *B. napus* plants. Photo credits—Watanabe Seed Co., Ltd., Miyagi, Japan, and Jon West (white rust and blackleg), Rothamsted Research, UK.

Several biotic factors, including fungi, bacteria, viruses, pests, and weeds, cause drastic yield losses of *Brassica* vegetables [2]. Biotic stress causes 20–30% of yield losses in *Brassica* vegetables resulting in substantial economic damage (Table 1) [3,4]. Diseases such as Fusarium wilt/Fusarium yellows (FY), clubroot, downy mildew (DM), black rot, soft rot, sclerotinia rot (SR), and turnip mosaic virus (TuMV) are among the most challenging biotic stresses to manage in *Brassica* vegetables (Figure 2, Table 1). Prevention of disease using integrated pest management, including crop rotation, cultivation and tillage practices, and biological control (e.g., *Coniothyrium minitans* against *Sclerotinia sclerotiorum*, arbuscular mycorrhizal fungi, and *Bacillus* species) is complex and often insufficient to fully control these diseases [5]. Management of pests or diseases by application of pesticides (insecticides or fungicides) is harmful to the environment and human health due to pesticide residues [5]. Therefore, breeding disease-resistant varieties is the most effective way to control diseases and increase the yield and quality of *Brassica* vegetables while minimizing the environment and human health risks.

**Table 1:** Prominent diseases of *Brassica* vegetables and associated crop losses.

Disease	Causative Agent	*Brassica* Species	Ideal Climatic Condition	Major Symptoms	Yield Losses	References
Fusarium wilt/yellow	*Fusarium oxysporum* f.sp. *conglutinans* (*Foc*) or *rapae* (*For*)	*B. rapa*; *B. oleracea*	16–35 °C, high soil moisture	Leaf yellowing, wilting, brown necrosis of the lower levels, stunted growth, and defoliation	Severe	[6]
Clubroot	*Plasmodiophora brassicae* (*Pb*)	*B. rapa*; *B. oleracea*	Acidic soil (pH < 6.8), wet and warm (>15 °C), low Ca and B, high ammonia	Wilting, stunting, and yellowing of shoots, club-shaped galls in roots	10–15%; 30–100% (severely infested fields)	[7,8,9]
Downy mildew	*Hyaloperonospora parasitica (Hp)*	*B. rapa*; *B. oleracea*	10–16 °C (germination and penetration of conidia), 20–24 °C (haustoria formation), high RH (≥85%)	Angular-shaped pale green to yellowish spots bound by leaf veins	*B. rapa*: ~90% damage of outer leaves; *B. oleracea*: 10–34% in cauliflower (20–35% seed crop); 16–20% in cabbage (50–60% seed crop)	[8,10,11]
Black rot	*Xanthomonas campestris* var. *campestris (Xcc)*	*B. oleracea*	>20 °C, >60% RH	‘V-shaped’ yellow-colored lesions with blackened veins, necrotic	10–50%, 60% in susceptible variety	[8,11]
Sclerotinia rot or Stalk rot	*Sclerotinia sclerotiorum*	*B. oleracea*	16–24 °C, >80% RH, cool and moist, >10 °C soil temp	Foliage Brassica: white fungal growth and small black sclerotia; Head: watery soft rot	17% seeds in cauliflower	[8,12,13]
Soft rot	*Pectobacterium carotovorum (Erwinia carotovorum)*	*B. rapa*; *B. oleracea*	Prolonged moisture, high RH, mild temperatures (21–25 °C), low Ca	Yellow-brown leaves, rotted leaves	Severe losses (25–40%)	[14]
Alternaria leaf/blight or black spot	*Alternaria brassicae* and *A. brassicicola*	*B. rapa*; *B. oleracea*	18–30 °C, ~90% RH	Pale to dark brown circular and zonate leaf spots	20–80%, 59% in seed crop	[8,15,16]
Blackleg/stem cankers	*Leptoshaeria maculans*	*B. rapa*; *B. oleracea*	5–20 °C (grows well up to 32 °C), low pH, wet climate	White to pale/dark brown spots on leaves, cankers in the stem	30–50%	[8,17,18,19]
White rust/blister	*Albugo candida*	*B. rapa*; *B. oleracea*	16–25 °C, additional moisture after rainfall at the dryland	Upper surface of leaves: Yellow spots Lower surface of leaves: small, white, blister-like pustules; necrosis, leaf curling, defoliation, and stunted growth	Up to 60%	[20,21,22]
Turnip mosaic	Turnip mosaic virus (TuMV)	*B. rapa*, *B. oleracea*	22–30 °C	Mottling and necrotic spots, ring spots, leaf distortion, and leaf yellowing	As high as 65–70%	[11,23]

pH—negative logarithm (base 10) of H^+^ concentration; Ca—calcium; B—boron; RH—relative humidity.

Understanding the genetic and epigenetic landscapes of disease resistance is crucial for *Brassica* vegetable breeding. Genetic studies reveal the blueprint of disease resistance, where DNA markers linked to the resistance (*R*) genes are critical tools in breeding programs and widely applied for marker-assisted selection (MAS) to control diseases in *Brassica* vegetables [24]. Epigenetic studies disclose the finely tuned transcriptional regulation responding to the environmental signal, contributing to resistance mechanisms [25]. Studies of epigenetic transcriptional regulation—gene activation or silencing—mediated by DNA methylation/demethylation or histone modifications (acetylation, methylation, phosphorylation, or ubiquitination) make valuable contributions to molecular breeding of *Brassica* vegetables [26]. Specifically, a clear understanding of the epigenetic mechanisms underlying disease resistance is needed for resistance breeding. Molecular studies can also help understand host–pathogen interaction mechanisms to identify *R* genes, to develop *R* gene-linked DNA markers, and to understand epigenome mechanisms for disease resistance in *Brassica* vegetables.

Diverse agroecological regions with varying environmental factors influence the genetic behavior of both crop plants and pathogens, making it difficult for resistance breeding in the context of a globally changing climate. Moreover, the race or strain specificity of pathogen species also varies across agroecological zones, adding another layer of complexity for breeding of disease resistance. This review summarizes the progress in genetic and epigenetic studies on biotic stress in *Brassica* vegetables. We aim to clarify the molecular mechanisms behind biotic stress resistance to develop innovative breeding strategies for boosting future productivity in *Brassica* vegetables.

## 2. Defense Mechanisms and Host–Pathogen Interaction in Plants

Plants use pattern-triggered immunity (PTI) and effector-triggered immunity (ETI) to defend against pathogens [27]. Pattern-recognition receptors (PRRs), localized at the plasma membrane, detect pathogens/microbes via pathogen-associated molecular patterns (PAMPs)/microbe-associated molecular patterns (MAMP) or damage-associated molecular patterns (DAMPs) [28,29,30,31]. PAMPs/MAMPs activate PTI; however, pathogens/microbes can secrete effectors into host cells or into the apoplast to suppress PTI activation [31]. PRRs activate a conserved set of defense responses, including transcriptional reprogramming, plant cell wall reinforcement and hormonal changes, and production of antimicrobial compounds [32]. Plants lacking PRRs are generally more susceptible to pathogens, highlighting the essential role of PRRs in PTI and disease resistance [33,34]. Nucleotide-binding (NB) leucine-rich repeat (LRR) receptors (NLRs) detect intracellular pathogen effectors and initiate ETI [35,36]. ETI frequently triggers hypersensitive responses (HR), leading to programmed cell death, increased salicylic acid (SA) synthesis, and expression and/or activation of *R* genes. *R* genes encode with intracellular NLRs or transmembrane surface receptors, i.e., receptor-like kinases (RLKs) or receptor-like proteins (RLPs) [37,38]. NLRs encode proteins with N-terminal Toll/Interleukin-1 receptor (TIR) or coiled-coil (CC) domains, along with an NB-ARC domain (previously referred to nucleotide-binding site (NBS)), and an LRR domain (TIR–NBS–LRR or CC–NBS–LRR). The recognition of specific effectors by corresponding R proteins is the basis for the gene-for-gene resistance model [39]. Plants also exhibit systemic acquired resistance (SAR), which allows faster and stronger activation of a wide range of defense responses, and often operates independently of pathogen specificity. SAR protects uninfected parts from subsequent attacks that are distant to the initial infection site. SAR is induced by systemic immune signals including proteins, lipids, SA, and other hormones that promote pathogenesis-related (*PR*) gene expression [40]. However, *PR* genes like *PR1* are also locally induced. SA, ethylene (ET), and jasmonic acid (JA) play a central role in the regulation of *PR* gene expression to initiate SAR [41].

Two copies of genes, encoding a bacterial flagellin-sensing receptor (FLS) and an elongation factor-Tu receptor (EFR), are present as PRR homologs in *B. rapa*, but only one copy of each gene is functional [42]. *B. rapa* consists of a less fractioned (LF) and two more fractioned (MFs: MF1 and MF2) subgenomes. The functional *FLS* gene, *BraFLS2* (Bra017563), is located in the MF2 subgenome, and the functional *EFR* gene, *BraEFR2* (Bra002305), is in the LF subgenome [42]. In *B. rapa*, the SA signaling pathway contributes to resistance against *Fusarium oxysporum* [43,44] and *Albugo candida* [45], while both SA and JA signaling pathways contribute to resistance against *Plasmodiophora brassicae* [46]. The JA and ET signaling pathways are crucial for resistance against necrotrophic pathogens such as *Alternaria brassicae* and *A. brassicicola* [47,48,49,50,51,52,53]. These findings suggest that, as in other plants, the SA, ET, and JA signaling networks collectively contribute to the activation of defense response against diverse pathogens/microbes in *Brassica* vegetable plants.

The level of host plant defense against diseases depends on specific interactions between host plants and pathogens/microbes. Plants have intimate interactions with pathogens/microbes; sometimes these interactions are beneficial, resulting in symbiotic associations, and other times, interactions are harmful, resulting in parasitic associations [54]. Next-generation sequencing (NGS) and omics approaches have been applied to various *Brassica* vegetables to understand their interactions with pathogens in *B. rapa* and *B. oleracea* [55,56,57]. Transcriptome analysis following infection with *P. brassicae* (hereafter *Pb*) revealed 32 upregulated and 16 downregulated genes in this plant–pathogen interaction [46]. Pangenomics have been developed in *B. rapa* [58] and *B. oleracea* [59], identifying novel candidate *R* genes and developing molecular markers that enhance the speed and precision of breeding program by broadening the *Brassica* gene pool. Host–pathogen interconnections have been shown within the *A. candida* and *Hyaloperonospora brassicae* pathosystems of *B. rapa* [45,60], and the fungal *S. sclerotiorum* and bacterial *Xanthomonas campestris* pathosystems of *B. oleracea* [61,62]. Bol020547, Bol028392, and Bol045724 encoding copies of *cytokinin dehydrogenase/oxidase* (*CKX*) were significantly upregulated in *B. oleracea* CR line, suggesting their role in this host–pathogen interaction [63]. The developmental stage of host plants (e.g., cotyledon, seedlings, rosette, and mature stages) and environmental conditions (e.g., temperature, relative humidity, CO_2_ concentration, soil pH, soil moisture, and soil nutrients) directly influence disease severity, which also depends on the race or pathotype of the pathogen species capable of triggering different levels of severity across geographic regions. Resistant varieties, which carry *R* genes against a specific race or pathotype, can be susceptible to other races or pathotypes. Pathogens or microbes can overcome host resistance capacity through the evolution of virulence causing high disease severity in previously resistant host plants [64]. Therefore, crop rotation using resistant varieties with different or alternative *R* genes is a strategy to minimize unexpected losses of *R* gene efficiency.

## 3. Host Resistance: Genomic Loci, Molecular Markers, Candidate Genes, and Transcription

*Brassica* vegetables have diverse phenotypes, for which, many single-nucleotide polymorphisms (SNPs) have been used as molecular markers in breeding. Distinctness, uniformity, and stability are necessary for new variety, and breeding with the help of molecular markers can ensure those. Various types of molecular markers including amplified fragment length polymorphisms (AFLP), inter-simple sequence repeat polymerase chain reaction (ISSR), simple sequence repeats (SSR), cleaved amplified polymorphic sequences (CAPS), sequence-characterized amplified region (SCAR), kompetitive allele-specific PCR (KASP), SNP, and insertion–deletion (InDel) polymorphisms have enabled rapid and precise analyses of germplasm evaluation, trait mapping, genetic mapping, quantitative trait locus (QTL) identification, genetic diversity analysis, MAS, and manipulation in breeding [65,66,67,68,69,70,71].

Molecular mapping of disease resistance genes is a critical prerequisite for effective resistance breeding in *Brassica* vegetables and also supports analysis of other beneficial traits, such as growth habit, yield, and flowering time [5,72]. SNPs are the most abundant and have been widely used for resistance breeding programs in *B. rapa* and *B. oleracea* [65,73,74,75,76,77]. Polymorphic InDel markers have great value for genetic analysis, construction of linkage maps, and MAS in *Brassica* vegetables [78,79,80]. NGS technologies such as genotyping-by-sequencing (GBS), QTL-seq, bulked segregant analysis sequencing (BSA-seq), genotyping by random amplicon sequencing–direct (GRAS-Di), and bulked segregant RNA sequencing (BSR-seq) are increasingly used for DNA marker development, as well as QTL and gene identifications.

### 3.1. Clubroot

#### 3.1.1. QTL Mapping in *B. rapa*

**Figure 3:**
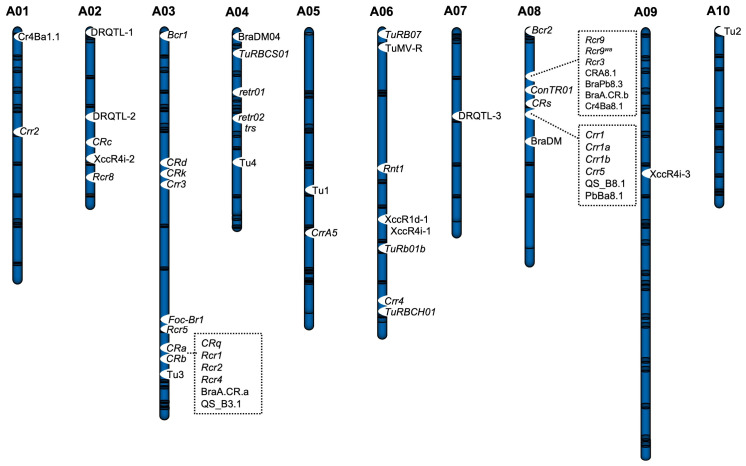
Distributions of major disease-resistant QTLs across A01-A10 chromosomes of *B. rapa* (revised and expanded from Figure 2 in [6]). The figure shows the clustering of resistance genes within particular genomic regions, especially at the bottom of A03 and the top of A08. CR loci: *Crr1*, *Crr1a*, *Crr1b*, *Crr2*, *Crr3*, *Crr4*, *Crr5*, *CrrA5*, *Bcr1*, *Bcr2*, *Rcr1*, *Rcr2*, *Rcr3*, *Rcr4*, *Rcr5*, *Rcr8*, *Rcr9*, *Rcr9^wa^*, *Rcr1*, *Rcr2*, *Rcr4*, *CRa*, *CRb*, *CRc*, *CRd*, *CRk*, *CRq*, *CRs*, QS_B3.1, QS_B8.1, PbBa8.1, BraA.CR.a, BraA.CR.b, Cr4Ba1.1, and Cr4Ba8.1. YR loci: *Foc-Br1* (*Foc-Br1a* and *Foc-Br1b*). Downy mildew-resistant loci: BraDM and BraDM04. Black rot-resistant loci: XccR1d-1, XccR4i-1, XccR4i-2, and XccR4i-3. Turnip mosaic virus (TuMV) disease resistance loci: *retr01*, *retr02*, *ConTR01*, *Rnt1*, *trs*, *TuRBCH01*, *TuRBCS01*, *TuRB07*, *TuRB01b*, *TuMV-R*, Tu1, Tu2, Tu3, and Tu4. DRQTL-1, DRQTL-2, and DRQTL-3.

*Pb* is the causal agent of clubroot disease in *Brassica* vegetables, and there are multiple pathotypes and isolates. Resistance mechanisms to specific pathotypes may not confer resistance to others. Over 32 major clubroot resistance (*CR*) genes have been identified in *B. rapa* vegetables, including *Crr1*, *Crr2*, *Crr3*, *Crr4*, *Rcr8*, *Rcr9*, *CrrA5*, *Rcr1*, *Rcr2*, *Rcr4*, *CRa*, *CRb*, *CRd*, *CRk*, and *CRs*, and their linked DNA markers have been developed (see these gene positions in Figure 3, Table 2) [6,81]. BSA-Seq combined with genetic mapping in an F_1_ population (500 plants for primary mapping and 3290 plants for fine mapping) of a cross between DW (resistant/heterozygous *CR* genes) and HZSX (susceptible) lines identified two *CR* loci, CRA8.1a and CRA8.1b, on chromosome A08 [82]. The CRA8.1b locus is responsible for resistance against *Pb* isolates from Zhijang of Hubei and Xinmin of Liaoning provinces of China. CRA8.1a and CRA8.1b loci together confer resistance against two different *Pb* isolates from the Xinmin region [82]. Two QTLs were identified on chromosomes A01 and A08 using BSA-seq; six genes on A01 (Bra013275, Bra013299, Bra013336, Bra013339, Bra013341, and Bra013357) and one gene on A08 (Bra020861) were suggested as candidates for *CR* genes [83]. The InDel marker, Crr1-196, was able to precisely differentiate between resistant and susceptible genotypes [83]. The BraPb8.3 locus was identified within a 173.8 kb region on chromosome A08, flanked by the markers srt8-65 and srt8-25, as contributing to CR in Chinese cabbage. Within this region, Bra020861 (encoding a TIR-NBS-LRR domain containing protein) and Bra020876 (encoding an LRR domain containing protein) genes were identified as candidates of *CR* genes [84]. Notably, Bra020861 was identified as a candidate of *CR* gene by two different research groups. Another *CR* locus, CRA3.7, was mapped on chromosome A03 in Chinese cabbage using an F_2_ population derived from a cross between a line harboring CRA3.7 and a susceptible inbred line [85]. The syau-InDel3008 marker was closely linked to the CRA3.7 locus. Among 54 TIR-NBS-LRR encoding genes in the QTL region, Bra019376, Bra019401, Bra019403, and Bra019410, were highly expressed in CR lines, suggesting these genes are candidates for the CRA3.7 locus [85]. The *Crr5* gene was mapped in 78.95 kb (19,774,426–19,853,376 bp) region on chromosome A08, flanked by DNA markers Su1-seq1 and Crr5-K35, using BSA-seq and KASP markers using resistant- and susceptible-pools of a *B. rapa* F_2_ population [86]. A TIR-NBS-LRR encoding gene, DH40A08G013380 (homologous to AT5G11250 in *Arabidopsis thaliana*), was identified within this interval. Two Crr5-specific KASP markers (Crr5-funK3 and Crr5-funK4) were developed for precise MAS [86].

The loss of the 172 amino acids at the C-terminal (fragment 2) region of Crr1a abolishes clubroot disease in Chinese cabbage, demonstrating the functional importance of the LRR domain [87]. Variety-specific insertions detected in exon 1 and exon 4 of *Crr1a* enabled the development of allele-specific markers capable of distinguishing between functional and non-functional *Crr1a* alleles [87]. Two insertions and one deletion in the *CRa* gene were also found in the exon 4 of resistant and susceptible Chinese cabbage lines. Co-dominant InDel markers, CRaEX04-1 (fragment size is 321 bp in the resistant line and 704 bp in the susceptible line) and CRaEX04-3 (fragment size is 704 bp in the resistant line and 413 bp in the susceptible line), can successfully differentiate between resistant and susceptible lines [88]. Using BSA-seq and KASP analysis, the CRA8.1.6 locus was mapped on chromosome A08 in turnip, and a candidate gene BraA08g015220.3.5C was identified [89]. A total of 249 SNPs, 7 insertions, 6 deletions, and a 5310 bp LTR retrotransposon within the first intron (at 909 bp) of BraA08g015220.3.5C was detected in disease-resistant material BrT18-6-4-3. This LTR retrotransposon was absent in the susceptible line, while the LTR insertion was present in the other susceptible lines, suggesting it is not associated with clubroot susceptibility. In contrast, the susceptible line carried an insertion and two deletions in its exon 4, which caused a frameshift mutation at position 8551 bp and premature termination of C-terminal translation within the LRR domain [89]. By contrast, the *CRA8.1.6* candidate gene showed 99.4% sequence identity with *Crr1a*, and is an allelic variant of *Crr1a* conferring CR in turnip. An InDel marker (CRA08-InDel) and a KASP marker (CRA08-KASP1) efficiently distinguished genotypes with clubroot resistance and susceptibility [89]. The *CRq* gene was identified on chromosome A03 by BSA-seq using an F_2_ population derived from double haploid (DH) lines with clubroot resistance and susceptibility [90]. Sequence analysis showed a 72 bp insertion in the exon 3 of the *CRq* gene in the susceptible line that resulted in the loss of resistance by disruption of the LRR region [90].

F_2_ populations from two different crosses between a clubroot-resistant turnip line (*B. rapa* subsp. *rapifera* ECD 02, resistance to Canadian *Pb* isolates) and two *B. rapa* accessions susceptible to clubroot were studied [91]. A phenotypic segregation ratio (15 resistant:1 susceptible) was observed against 3H, 5X, and 5G pathotypes of *Pb*. Two major *CR* genes, *CRa/CRb^Kato^* on chromosome A03 and *Crr1* on chromosome A08, were identified as conferring resistance to the 5X and 5G pathotypes of *Pb* in both F_2_ populations. Segregation ratios and molecular analyses confirmed the inheritance of *CRa/CRb^Kato^* and *Crr1* genes and epistatic effects between these two major genes [91,92]. BC_1_S_1_ lines were developed from a cross between *B. rapa* canola ACDC (susceptible) and turnip ECD02 (resistant) where the F_1_ populations were resistant to the 3A, 3D, 3H, and 5X pathotypes of *Pb* [93]. A total of 219 genes were detected within a single co-localized QTL (*Rcr9*), among which four genes (BraA08g013630.3C, BraA08g013130.3C, BraA08g012920.3C, and BraA08g012910.3C) encode R proteins [93].

Two QTLs on chromosomes A03 and A08 were identified from an F_2_ population derived from a cross between a resistant turnip line and a susceptible Chinese cabbage line using BSA-Seq. These loci were further narrowed down using an F*_3_* population and KASP markers [94]. Three candidate *R* genes on chromosome A03 (Bra006630, Bra006631, and Bra006632), and two on chromosome A08 (Bra030815 and Bra030846), all encoding TIR–NBS–LRR protein were proposed as potential *CR* genes [94]. The PbBrA08^Banglim^, a single dominant QTL of *Pb* pathotype “Banglim”, was located near *Crr1*, *CRs*, and *Rcr9* on chromosome A08 in a *B. rapa* DH F_2_ population. The flanking marker (09CR.11390652) precisely differentiates between resistant and susceptible genotypes [95]. The *Rcr3* and *Rcr9^wa^* genes were mapped on chromosome A08 against *Pb* pathotypes 3H and 5X, respectively, using BSR-Seq and KASP markers [96]. *Rcr3* candidates were Bra020951, Bra020974, and Bra020979 genes, while *Rcr9^wa^* candidates were Bra020827, Bra020828, and Bra020814 genes [96]. Rutabaga (*B. napus* ssp. *napobrassica*) accessions from Norway, Sweden, Finland, Denmark, and Iceland were used for the CR loci identifications with 17 isolates from 16 pathotypes of *Pb* [97]. Genomic regions on chromosome A03 and A08 were detected as *Pb* pathotype resistance hotspots. The CR hotspot on chromosome A03 coincided with the locations of *CRa*, *CRb*, and *Rcr1* genes, while the hotspot on chromosome A08 was near the *Crr1* gene [97]. Using BSR-Seq on resistant and susceptible bulks against 17 isolates from 16 pathotypes of *Pb*, seven novel major QTLs were identified. These included four QTLs on chromosome A08, and one each on chromosomes A05, C01, and C07 [98].

#### 3.1.2. QTL Mapping in *B. oleracea*

**Figure 4:**
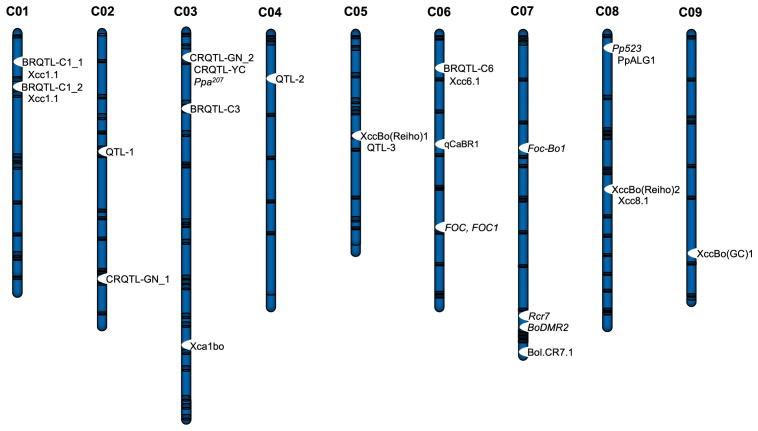
The C genome (chromosomes C01-C09) of *B. oleracea* shows the distribution of major disease-resistant QTLs. CR loci: *Rcr7*, CRQTL-C1_1, CRQTL-C1_2, CRQTL-GN_1 CRQTL-GN_2, CRQTL-YC, and Bol.CR7.1; YR loci: *FOC*, and *FOC1*; Downy mildew-resistant loci: *BoDMR2*, *Ppa523*, PpALG1, and *Ppa^207^*; Black rot-resistant loci: QTL-1, QTL-2, QTL-3, Xca1bo, XccBo(Reiho)1, XccBo(Reiho)2, XccBo(GC)1, BRQTL-C1_1, BRQTL-C1_2, BRQTL-C3, BRQTL-C6, Xcc1.1, Xcc6.1, Xcc8.1, and qCaBR1.3.1.3. Introgression of *CR* genes using molecular markers.

Some *CR* genes governing QTL in *B. oleracea* (cabbage, cauliflower, and broccoli) confer complete but race-specific resistance to clubroot disease. Since the first CR QTL was mapped in broccoli against *Pb* physiological race 7 (PR7) [99], many CR QTLs have been identified in *B. oleracea* (Figure 4, Table 2). Two QTLs were identified in kale against ECD 16/31/31 [100], and two QTLs in cabbage against ECD 16/3/30 [101]. Using SNP-based techniques, nine QTLs conferring resistance to PR1, PR2, PR4, and PR7 pathotypes were identified in kale [102], and twenty-three QTLs conferring resistance to PR4 pathotype were identified in cabbage [103]. Association mapping has identified ten QTLs against pathotypes 3A and 5X-LG2 in *B. oleracea* accessions [104]. In cabbage, two QTLs (CRQTL-GN_1 and CRQTL-GN_2) on chromosomes C02 and C03 against PR9 pathotypes and one QTL (CRQTL-YC) on C03 against PR2 were identified by GBS [105]. Using QTL-seq, four QTLs (one on C04 and three on C07) were identified in cabbage against PR4 pathotype, with two candidate genes (Bol037115 and Bol042270) [106]. Using BSA-seq, Bol.CR7.1 locus was identified in C07 of cabbage against PR4 pathotype which was fine-mapped and a potential CR gene (*Bol.TNL.2*) was identified [107]. A major QTL, pbBo(Anju)1, against PR4 was identified in cabbage (cv. Anju) [108], along with four minor QTLs, pbBo(Anju)2, pbBo(Anju)3, pbBo(Anju)4, and pbBo(GC)1 [108,109]. Two major QTLs, Rcr_C03-1 and Rcr_C08-1, located on chromosomes C03 and C08 of *B. oleracea*, respectively, were identified via GBS [110]. Rcr_C03-1 harboring ten TIR-NBS-LRR encoding genes confers resistance to eight *Pb* pathotypes (2B, 5C, 5G, 3H, 8J, 5K, 5L, and 3O) and Rcr_C08-1 harboring one TIR-NBS-LRR encoding gene conferred resistance against two *Pb* pathotypes (8J and 5K) [110]. Chromosomes C03 and C08 in *B. oleracea* have high synteny with chromosomes A03 and A08 in *B. rapa*, respectively [111]. CNL class proteins Boc08g03058.1 (homologous to AT1G12290.1 in *A. thaliana*), Boc08g03059.1 (AT1G12220.2), Boc08g03179.1 (AT1G53350.1), and Boc08g03180.1 (AT1G53350.1) were identified as candidates for Rcr_C08-1 [110]. The syntenic relation between chromosomes A03/A08 of *B. rapa* and C03/C08 of *B. oleracea* highlight existence of conserved genomic regions controlling clubroot resistance across the species.

Linked markers are identifiable DNA sequences at specific physical locations which can be used to select plants for targeted traits (known as marker-assisted selection (MAS)). In *Brassica* vegetables, linked markers for CR loci can be used for MAS and/or gene pyramiding (gene pyramiding—combining multiple genes into a single variety) to develop resistant varieties. A limited number of varieties highly resistant to clubroot have been developed, suggesting the possibility of future MAS breeding of CR varieties. In Chinese cabbage, lines homozygous for *CRa* (SC2930 marker), *CRk* (HC688 marker), and *CRc* (B50 marker) genes through MAS exhibited high resistance to six field *Pb* isolates [112]. The *CRb* gene was introgressed using linked markers, TCR74 and TCR79, and a newly developed line from the BC_4_F_2_ population showed no disease incidence-related *Pb* pathotype 4 [113]. Chinese cabbage lines accumulating homozygous alleles of *CRa* and *CRd* genes also exhibited enhanced resistance against six *Pb* isolates compared to parental lines [114]. A major *CR* gene (*CCR13685* QTL) was introduced into pak choi using the K-3 marker, resulting in significantly improved resistance to clubroot [115]. A *Crr5* gene-specific KASP marker (Crr5-funK3) was used to introgress the *Crr5* gene from the resistant donor into a susceptible line, leading to the development of a near-isogenic line carrying the complete DNA fragment of *Crr5* obtained through marker-assisted backcrossing [86].

Like *B. rapa*, a single *CR* gene with strong resistance has not yet been identified in *B. oleracea* despite the discovery of over 50 QTLs [6]. Accumulation of a major *CR* gene at the PbBo(Anju)1 locus along with minor *CR* genes at the PbBo(Anju)2, PbBo(Anju)3, and PbBo(Anju)4 loci enhanced resistance against six *Pb* isolates in *B. oleracea* [109]. *Rcr7* located on LG7 was considered as a major *CR* gene in cabbage (cv. Tekila and cv. Kilaherb) [116]. Due to the limited resistance sources in *B. oleracea*, researchers have introduced *CR* genes from *B. rapa* into *B. oleracea*. The *CRa*, *CRb*, and *Pb8.1* genes from *B. rapa* were introgressed into cabbage (*B. oleracea* var. *capitata*), and BC_2_ populations carrying all three *CR* genes showed resistance against race 4 of *Pb* [117]. The introgression of *Crr1*, *Crr2*, *Crr3*, *CRa*, *CRb*, and *CRc* genes of *B. rapa* into cabbage has also conferred strong resistance against the clubroot pathogen [118].

#### 3.1.3. Transcriptome Analysis for CR Gene

Genes whose expression is altered by *Pb* infection are being explored. From gene ontology (GO) analysis of the RNA-seq data, genes involved in ‘metabolic pathway’, ‘plant–pathogen interaction’, ‘plant hormone signal transduction’, ‘biosynthesis of secondary metabolites’, and ‘phenylpropanoid biosynthesis pathway’ tended to be altered following *Pb* infection [46,119,120,121,122]. A CR Chinese cabbage variety, ‘Akimeki’, exhibited susceptibility to several Korean *Pb* pathotypes; therefore, its transcriptome profile was compared to mock-inoculated and inoculated samples using two Korean *Pb* strains (Seosan-susceptible and Hoengseong2-resistant) [123]. Genes upregulated by Seosan inoculation showed an enrichment of categories related to defense response and JA regulation. The expression of Bra004873 (an SA pathway gene) and Bra018271 (a JA pathway gene) was two-fold higher following *Pb* inoculation, while another JA pathway gene (Bra040746) was expressed two-fold less [46]. Activation of genes involved in hormone signaling and cell wall metabolism were involved in *Pb* resistance mechanisms of Chinese cabbage [124]. Genes associated with PTI and ETI were altered following *Pb* infection and the expression patterns of genes involved in the JA and SA signaling pathways following infection were opposite between resistant and susceptible lines, suggesting that crosstalk between SA and JA signaling pathways is important for the defense response against *Pb* [123]. Alternative splicing (AS) events may also be involved in post-transcriptional mechanisms to contribute to the fine-tuning of disease resistance. Genes involved in SA and JA signaling pathways undergo AS events to regulate resistance mechanisms. An AS event was found in 1201 genes of a CR Chinese cabbage line using PacBio RS II SMRT sequencing, where six genes—one related to disease resistance (BraA07g042230.3C) and five associated with defense response (BraA02g025510.3C, BraA02g025540.3C, BraA06g025400.3C, BraA06g040100.3C, and BraA03g042180.3C)—were differentially expressed, suggesting their potential involvement in resistance mechanisms [125].

Transcriptomes were compared between *Pb*-resistant and susceptible root samples of *B. oleracea*, and Bol010786 (CNGC13) and Bol017921 (SD2-5) CR candidate genes were identified [126]. Galled and symptomless roots of the same plants also showed differences in gene expression patterns with upregulation of genes related to cell wall synthesis and reinforcement occurring in symptomless roots [127]. This might be due to changes in hormone metabolisms, including downregulation of JA biosynthesis, upregulation of SA-mediated defense responses, and increased cytokinin metabolism and signaling in symptomless roots [127]. ETI and PTI pathways were involved in *Pb* resistance mechanisms of *Brassica* vegetables [126,128]. Defense-related *PR* genes, *WRKY28*, ethylene signaling transduction genes and ABA signaling genes may also be involved in *Pb* defense mechanisms in *B. oleracea* [128]. Differentially expressed genes (DEGs) at 7 and 21 days after *Pb* inoculation in cabbage enriched in plant–pathogen interaction where WRKY genes (BolC02g057640.2J, BolC09g006890.2J), LRR-domain genes (BolC02g013230.2J, BolC06g006490.2J), a disease resistance gene (BolC03g052660.2J), mitogen-activated protein kinase—MAPK gene (BolC07g052580.2J), and NAC (NAC is the *NAM*—*NO APICAL MERISTEM*, *ATAF1/2*—*ARABIDOPSIS TRANSCRIPTION ACTIVATOR FACTOR*, and *CUC2*—*CUP-SHAPED COTYLEDON* gene family) domain containing gene (BolC04g044910.2J) were involved in the defense response. On the other hand, microRNAs (miRNAs) such as_miR80 (BolC05g028200.2J) and miR139 (BolC02g008640.2J) interacted with mRNAs in response to *Pb* infection in cabbage [129].

**Table 2:** CR loci in *Brassica* vegetables (Adapted from Mehraj et al., 2020 [6]).

Parents	Pop.	Race or Pathotype	System	Chr.	Resistance Gene/Loci	Flanking or Linked Markers	PL (Mb)	PV (%)	Candidate Gene IDs	Ref.
***B*** ***. rapa***										
T136-8 (R), Q5 (S)	F_2_	Pb2	RFLP	A03	*CRa*	HC352b~HC181	*-*	*-*	*-*	[130]
			SCAR			HC352b-SCAR	*-*	*-*	*-*	[131]
			SCAR			HC181-SCAR	*-*	*-*	*-*	[132]
94SK (S) and CR Shinki (R) (DH)	F_2_	Pb4	CAPS, SCAR	A03	*CRb*^(i)^	TCR09~TCR05	*-*	*-*	*-*	[133]
						TCR079~TCR108	*-*	*-*	*-*	[134]
T-line (R) and V-line (S)	F_2_	Pb14	SSR	A03	*CRb*^(i)^	KBrH059N21F~KBrH129J18R	21.16~24.76	*-*	*-*	[135]
			SSR, BAC, InDel			KB59N07~B1005	24.2~24.34	*-*	Bra019410, Bra019413 ^a^	[136,137]
SG (R) and BJN3 (S)	F_2_, F_3_	Pb4	SSR, UGMS	A03	QS_B3.1 ^(A)^	sau_um028~At4g35530	22.28~29.98	70.55	-	[138]
G004 (R) and A9709 (S), DH lines	F_2_	Pb4	SSR	A08	*Crr1*	BRMS-088	*-*	*-*	*-*	[139]
		W01	RAPD, RFLP, SSR, InDel			BRMS-297~BRMS-088	*-*	26.8	*-*	[140]
		Ano-01					*-*	71.7	*-*	
		Pb5, Pb7, Pb9, Pb14	SSR		*Crr1a*	BSA7	*-*	*-*	*-*	[141]
			BAC-clones		*Crr1b*	AT27	*-*	*-*	*-*	
Five resistant hybrids	BC_3_F_2_	CanFI	SNP, SSR, SCAR	A03	*CRa*	M8~M10	24.26~24.45	-	-	[142]
SCNU-T2016 (R), CC-F920 (S)	F_2_	Pb4	SNP	A08	*CRs* ^(B)^	A08:8577582~A08:11505101	7.86–11.86	96.87	Bra020918, Bra020876 ^b^	[143]
SG (R) and BJN3 (S)	F_2_, F_3_	Pb4	SSR, UGMS	A08	QS_B8.1 ^(C)^	BRPGM0920~BRPGM0173	6.15	7.28	-	[138]
G004 (R) and A9709 (S), DH lines	F_2_	Pb4	SSR	A01	*Crr2*	BRMS-096	*-*	*-*	*-*	[139]
		W01	RAPD, RFLP, SSR, InDel			BRMS-100~BRMS-096	*-*	18.3	*-*	[140]
N-WMR-3 (R) and A9709	F_2_, F_3_	Pb4	STSs	A03	*Crr3*	OPC11-2S	*-*	-	*-*	[144]
			STSs, CAPS			BrSTS-33~BrSTS-78	*-*	-	*-*	[145]
20-2cc1 (R), EC-1 (S)	BC_1_	-	RAPD, SSR, SCAR, InDel	A03		BrID10041~BrID10031	*-*	-	*-*	[146]
G004 (R) and A9709 (S), DH lines	F_2_	W01	RAPD, RFLP, SSR, InDel	A06	*Crr4*	BN288D~WE24-1	*-*	10.5	*-*	[140]
		Ano-01					*-*	15.9	*-*	
DH40 (R, DH) and ECD01	F_2_	Pb4	SNPs, InDel, KASP	A08	*Crr5*^(B)^	Su1-seq1~ Crr5-K35	19.77~19.85		DH40A08G013380 ^c^	[86]
20-2cc1 (R), EC-1 (S)	BC_1_	-	RAPD, SSR, SCAR, InDel	A05	CrrA5	tau_cBrCR404~BrID10131	*-*	-	*-*	[146]
DingWen (R), HuangZiShaXun (S)	F_1_	PbXm, PbCd, PbZj, PbTc, and PbLx	SNPs, InDel	A08	CRA8.1a	A08-4346~A08-4624	10.7~11.5	-	BraA08g039174E, BraA08g039175E, BraA08g039193E ^c^	[82]
					CRA8.1b	A08-4624~A08-4853	11.5~11.9	-	BraA08g039211E, BraA08g039212E ^b^	
C9 (R) and 6R (S), DH lines	F_2_	K04	AFLP, RAPD, RFLP, STS, and SSR	A02	*CRc*	E14M3-02~m6R	-	68.5~72.1	-	[147]
K10 (R) and Q5 (S), DH lines	F_2_	M85, K04		A03	*CRk*^(Up1)^	HC688~OPC11-2S	-	50.2~71.1	-	
85-74 (R) and BJN3-1 (S)	F_2_, F_3_	Pb4	SNPs	A03	*CRd*^(Up1)^	yau389~yau376	15.03~15.09	-	Bra001160, Bra001161, Bra001162, Bra001175 ^b^	[148]
Y635-10 (R) and Y177-47 (S), DH lines	F_2_	Pb4	SNPs, InDels, SSR	A03	*CRq*^(i)^	GC30-FW/RV~BGA06	24.35~24.43	-	Bra019409, Bra019410, Bra019412, Bra019413 ^b^	[90]
ECD04 (R) and C59-1 (S)	BC_1_F_1_	Pb2, and Pb7	SSR	A01	PbBa1.1	cnu_m235a~hri_mBRMS056a	-	13.2, 18.7	-	[149]
		Pb2		A03	PbBa3.1	nia_m102a~sau_um034a	-	12.2	-	
		Pb10			PbBa3.2	cnu_m098a~sau_um516a	-	14	-	
		Pb7			PbBa3.3	cnu_m327a~cnu_m073a	-	18.70	-	
		Pb4		A08	PbBa8.1 ^(C)^	cnu_m490a~sau_um353a	-	35.20	-	
377 (R) and 12A (S)	F_2_	Pb4	SNPs, InDel	A08	BraPb8.3 ^(D)^	srt8-65~srt8-25	10.70~10.86	7.39	Bra020876, Bra020861 ^b^	[84]
Pak choy cv. FN (R) and ACDC DH line (S)	F_2_, BC_1_F_1_	Pb3	SSR, CAPS	A03	*Rcr1*^(ii)^	ms7-9~sN8591	24.26~24.50	96.50	Bra019409, Bra019410, Bra019412, Bra019413 ^b^	[150,151]
Chinese cabbage cv. Jazz (R) and ACDC (S)	F_1_	Pb3	SNPs, KASP	A03	*Rcr2*^(ii)^	SNP_A03_32~SNP_A03_67	24.14~24.39	-	Bra019409, Bra019410, Bra019412, Bra019413 ^b^	[152]
96-6990-2 (R) and ACDC (S)		Pb3H, Pb5x	SNPs, InDels, KASP	A08	*Rcr3*	A90_A08_SNP_M12 and M16	10.00 and 10.23		Bra020951, Bra020974, Bra020979 ^b^	[96]
T19 (R) and ACDC (S)	BC_1_S_1_	Pb2, Pb3, Pb5, Pb6, and Pb8	SNPs, InDels	A03	*Rcr4*^(ii)^	-	22.69~25.65	85~94	Bra012541, Bra019413, Bra019412, Bra019410, Bra019409, Bra019273 ^b^	[153]
PTWG (R) and ACDC (S)	BC_1_	Pb3	SNPs, InDels, KASP	A03	*Rcr5*^(Up2)^	SNP_A03_100~SNP_A03_83	23.47~23.34	-	-	[154]
T19 (R) and ACDC (S)	BC_1_S_1_	Pb5x	SNPs, InDels	A02	*Rcr8*	-	18.50~22.10	36.00	Bra022069, Bra022071, Bra026556, Bra032996 ^b^	[153]
		Pb5x		A08	*Rcr9*	-	7.11~13.59	39.00	Bra020936, Bra020861 ^b^	
96-6990-2 (R) and ACDC (S)	BC_1_	Pb3H, Pb5x	SNPs, InDels, KASP	A08	*Rcr9^wa^*^(E)^	A90_A08_SNP_M28 and M79	10.85 and 11.17		Bra020827, Bra020828, Bra020814 ^b^	[96]
ECD04 (R) and Yellow sarson (S)	BC_1_	CanFI	SRAP, SSR	A03	BraA.CR.a ^(ii)^	FSASS45b~FSASS79b	24.30~24.40	-	-	[155]
				A08	BraA.CR.b ^(E)^	S11R11~S08R08	10.78~10.93	-	-	
877 (R) and ‘255 (S)	F_2_, F_3_	Pb4	SNPs, KASP	A03	*Bcr1*	A03-1-192~A03-1-024	4.3~4.78	33.30	Bra006630, Bra006631, Bra006632 ^b^	[94]
				A08	*Bcr2*	A08-1-06~A08-1-705	0.02~0.79	13.30	Bra030815, Bra030846 ^b^	
Bap246 (R), Bac1344 (S)	F_2_	Pb4	SNPs, InDels	A01	Cr4Ba1.1	SNP-4678697~SNP-5170126	4.68~5.17	30.97	Bra013275, Bra013299, Bra013336, Bra013339, Bra013341, Bra013357	[83]
				A08	Cr4Ba8.1	A08-10700494~A08-10845219	10.70~10.85	8.65	Bra020861 ^b^	
***B. oleracea***										
EW (S), OSU CR-7 (R)	F_2_	Pb7	RFLP	1C	-	14a	-	-	-	
C10 (R), 48.4.7 (S)	F_2_	ECD	RAPD	-	2 QTLs	OPL6-780~OPB11-740, OPA16-510	-	-	-	[156]
Bi (R), Gr (S), DH lines	F_2_	ECD	RFLP, AFLP	LG3	Pb-3	4NE11a	-	-	-	[101]
				LG1	Pb-4	2NA8c	-	-	-	
Y2A, K269	F_2_	-	RAPD, RFLP	LG3	1 QTL	WG6A1~WG1G5	-	-	-	[157]
Tekila (R), Kilaherb (R) T010000DH (S)	F_1_	Pb3, Pb5x	KASP	C07	*Rcr7*	SNP_C7_44~SNP_C7_56	41~44	56–73	Bo7g108760, Bo7g109000 ^b^	
K269 (R), Y2A (S)	F_2_, F_3_	Km, Anno, Yuki	RAPD, RFLP, SCAR, CAPS	LG1	QTL1	SCA02a2	-	-	-	[158]
				LG3	QTL3	SCB50b~SCB74c	-	-	-	
				LG9	QTL9	SOPT15a~SCA25	-	-	-	
C10 (R), HDEM DH line (S)	F_3_	Pb1, Pb2, Pb4, Pb7	RAPD, RFLP	LG1	Pb-Bo1	Ae05.8800~T2	-	-	-	[102]
				LG2	Pb-Bo2	PBB38a~r10.1200	-	-	-	
				LG3	Pb-Bo3	Ae15.100~RGA8.450	-	-	-	
				LG4	Pb-Bo4	ELI3.983~aa9.983	-	-	-	
				LG5	Pb-Bo5a	PBB7b~ae05.135	-	-	-	
				LG5	Pb-Bo5b	ELI3.115~a18.1400	-	-	-	
				LG8	Pb-Bo8	C01.980~t16.500	-	-	-	
				LG9	Pb-Bo9a	Aj16.570~W22B.400	-	-	-	
				LG9	Pb-Bo9b	A04.1900~ae03.136	-	-	-	
Anju DH line (R), GC DH line (S)	F_2_, F_3_	PR4	SSR, CAPS	LG2	Pb-Bo(Anju)1	KBrH059L13	-	47.0	-	[108,109]
				LG2	Pb-Bo(Anju)2	CB10026	-	40.0	-	
				LG3	Pb-Bo(Anju)3	KBrB068C04	-	9.0	-	
				LG7	Pb-Bo(Anju)4	KBrB089H07	-	3.0	-	
				LG5	Pb-Bo(GC)1	CB10065	-	9.0	-	
C1220 (R), C1176 (S)	F_2_, F_3_	PR9	SNPs (GBS)	C02	CRQTL-GN_1	C2d-1(2)~C2g-1(1)	-	22.0~29.7	-	[105]
				C03	CRQTL-GN_2	C3a-1(11)~C3b-14(6)	-	23.5~29.1	-	
		PR2	SNPs (GBS)	C03	CRQTL-YC	C3a-1(11)–C3b-153(3)	-	47.1	-	
GZ87 (R), 263 (S)	F_2_	PR4	SNPs	-	23 QTL	-	-	6.1~17.8	-	[103]
W12 (R), Z5 (S)	-	Pb4	SNPs, InDels	C07	Bol.CR7.1	InDel_5177~InDel_519. R	51.77~51.94	-	BolC7t45647H (*Bol.TNL.2*)	[107]

^i^*Cra*, *CRb*, and *CRq* are co-localized; ^ii^ *CRq*, *Rcr1*, *Rcr2*, and *Rcr4* were co-localized with *Cra*; ^(A)^ Co-localized with *CRa*, *CRb*; ^(B)^ Co-localized with *Crr1a*; ^(C)^ Co-localized with *Crr1b*; ^(D)^ Co-localized with CRA8.1a; ^(E)^ Co-localized with *Rcr9*; ^(Up1)^ Upstream region of *Crr3*; ^(Up2)^ Upstream region of *CRa*/*CRb*. ^a^ NB-LRR encoded genes; ^b^ TIR-NBS-LRR encoded genes; ^c^ RLP encoded genes. Pop.—population; Chr.—chromosome; PL—physical location; PV—phenotypic variance; Ref.—reference/s; S—susceptible; R—resistant; DH—double haploid; F_1_, F_2_, and F_3_—first, second, and third filial generations; BC_1_, BC_2_, and BC_3_—backcrossed first, second, and third generation; BC_1_F_1_—first filial generation of the first backcross; BC_1_F_2_—second filial generation of the first backcross (comes from selfing of BC_1_F_1_); BC_1_S_1_—backcross segregating first generation; W01—Wakayama-01; CanF—Canadian field isolates; Km—Kamogawa; SNP—single-nucleotide polymorphism; RFLP—restriction fragment length polymorphism; SSR—simple sequence repeat; RAPD—random amplified polymorphic DNA; SRAP —sequence-related amplified polymorphism; CAPS—cleaved amplified polymorphic sequences; InDel—insertion–deletion; SCAR—sequence-characterized amplified region; GBS—genotyping-by-sequencing; AFLP—amplified fragment length polymorphism; KASP—Kompetitive Allele-Specific PCR; BAC—bacterial artificial chromosome; STS—sequence-tagged site; UGMS—unigene-derived reliable microsatellite; LG—linkage group.

### 3.2. Fusarium Yellows

Fusarium yellows (also known as Fusarium wilt) is caused by *Fusarium oxysporum f. sp. conglutinans* (*Foc*), which has two races, race 1 (Type A) and race 2 (Type B), and *F. oxysporum* f. sp. *rapae* (*For*). Of the two categories of YR (Fusarium yellows resistance), Type A resistance is temperature-independent and is controlled by a single dominant gene, while Type B resistance breaks down at 24 °C in *B. rapa* and *B. oleracea* [6]. Resistance to one race of *Foc* can be overcome by another race, and most studies on YR in *Brassica* vegetables have focused on race 1. The resistance mechanism against race 2 is controlled by one or more genes with additive, dominant, and epistatic modes of gene actions [159]. Further studies on race 2 resistance will aid breeders in developing non-race-specific YR varieties. Chromosomal loci containing *R* genes against *Foc* have been identified in *B. rapa* and *B. oleracea* (Table 3) [160,161,162,163,164,165,166,167].

Two genes encoding TIR-NBS-LRR, Bra012688, and Bra012689, were identified as candidate *R* genes for YR in *B. rapa*, and their closely linked markers map to *Foc* resistance in Chinese cabbage [160]. *Foc*-resistant lines of *B. rapa* vegetables show resistance to *For*, and the *For* resistance gene (*ForBr1*) map to the same locus as *FocBr1* on chromosome A03 [161]. In susceptible lines, there are six amino acid substitutions in *ForBr1*, and a DNA marker for *ForBr1* was tightly linked to the resistance phenotype, suggesting that *FocBr1* and *ForBr1* are the same *R* gene. Additionally, *FocBr1/ForBr1* and CR genes (*CRa* and *CRb*) are located in the same region on chromosome A03 but there is a physical distance between them (Figure 3), making it possible to develop varieties with dual YCR (Fusarium yellows and clubroot resistance).

In cabbage, the YR gene, *FocBo1*, was mapped to chromosome C07 and its closely linked marker, KBrS003O1N10, effectively differentiating susceptible and resistant lines [162]. Fine mapping of the *FocBo1* locus identified an orthologous gene (Bra012688) in *B. rapa*, suggesting the possibility of developing YR varieties with the help of *FocBo1* [163]. A *Foc* (race 1) resistance QTL was identified on chromosome C06 (it was later found to be on C07), flanked by M10 and A1 markers, and both markers effectively distinguish susceptible and resistant lines [164,165]. The re-Bol037156 gene encoding TIR-NBS-LRR in this QTL showed an InDel mutation (1 bp insertion and 10 bp deletion) in susceptible lines while resistant lines had no mutations, suggesting that the re-Bol037156 gene could be a candidate for *FocBo1* in cabbage [166]. Another SSR marker (Frg13) was identified being closely linked to the *FocBo1I* [167]. Subsequently, four markers (A1, M10, Frg13, and Ol10-D01) were tested in isogenic stable and unstable YR white cabbage lines [168]. The A1 marker did not show any allelic differences between stable and unstable lines, while M10 and Frg13 did. M10, Frg13, and Ol10-D01 were polymorphic and used for PCR analysis in an F_2_ segregating population. Only Ol10-D01 co-segregated with a 1:2:1 Mendelian ratio, indicating its potential utility as a DNA marker for YR breeding in cabbage varieties cultivated in southern Russia [168]. A set of DNA markers capable of distinguishing YR in *B. oleracea* was developed [169].

Activation by *Foc* inoculation of biosynthetic processes, such as SAR as well as JA-, ET-, and chitin-dependent pathways is involved in resistance mechanisms of *B. rapa* and *B. oleracea* [43,170]. In addition, SA-induced genes are involved in *Foc* resistance mechanisms of *B. rapa* [44]. From KEGG pathway analysis, NBS-LRR genes (*RPS4*, *RPS2*, and *CALM—Arabidopsis* homologs in *B. oleracea*) and *WRKY* transcription factor (TF) genes (*WRKY52* and *WRKY33*) were upregulated in a resistant line of *B. oleracea* at 3, 6, and 9 days post-inoculation with *Foc* (race 1) [171]. The upregulation of *ERF1* and *ERF2* in the resistant line suggests the involvement of the ET signaling pathway in the YR mechanisms in cabbage, similar to *B. rapa.* The expression levels of *JAZ1* (*JASMONATE-ZIM-DOMAIN PROTEIN 1*) and *TGA* (*TGA MOTIF-BINDING FACTOR*—a salicylic acid-responsive TF) increased with the duration of *Foc* inoculation in both the resistant and susceptible lines without significant differences in expression between the lines [171]. This study therefore suggests post-transcriptional or signaling-level regulation and more complex roles for JA and SA signaling pathways in *B. oleracea*.

**Table 3:** YR loci in *Brassica* vegetables (Adapted from Mehraj et al., 2020 [6]).

Parents	Population	Race	System	Chr.	Major Loci	Linked/Flanking Markers (PL in Mb) ^1^	Reference
***B. rapa***							
Chinese cabbage: RJKB-T21 and T23 (R), RJKB-T22, and T24 (S)	F_2_	Cong:1-1	RNA-seq	A03	*Foc-Br1a*	Bra012688m	[160]
					*Foc-Br1b*	Bra012689m	
***B. oleracea***							
Broccoli cv GCPO4 (S), Cabbage cv Anju (R)	F_2_	Cong:1-1	SSR	C07	QTL2 *(Foc-Bo1)*	KBrS003O1N10	[162]
	F_2_	Cong: 1-1	InDel	C07	*Foc-Bo1*	BoInd 2 and BoInd 11	[163]
Cabbage: 99–77 (R), 99–91 (S)	DH	FGL3-6	InDel	C06	*FOC*	M10 and A1	[164]
	DH, F_2_	FGL3-7	InDel	C06	*FOC1*	Bol037156 (38.8) and Bol037158 (38.8)	[166]
Cabbage: 01–20 (S), 96–100 (R)	DH	FGL3-6	InDel, SSR	C06	*FOC1*	Frg13	[167]
Raddish; YR RK15-1 (R), AKM (S)	F_2_	MAFF 731043 (*For*)	GBS, GRAS-Di	R07	*ForRs1*	AMP0000754~AMP0009342	[172]
				R02	*ForRs2*	AMP0010176~AMP0013639	

^1^ PL represents the physical location in megabase (Mb) that has been mentioned in the parenthesis in the linked marker column. S—susceptible; R—resistant; DH—double haploid; F_2_—second filial generations; SSR—simple sequence repeat; InDel—insertion–deletion; GBS—genotyping-by-sequencing; GRAS-Di—genotyping by random amplicon sequencing–direct.

### 3.3. Downy Mildew

Downy mildew (DM) is a disease that spreads primarily through air-borne spores of *Hyaloperonospora parasitica (Pers.)*. The disease first appears as a white cottony mass or powder on the lower leaves and later forms chlorotic irregularly shaped lesions on the upper leaves of various crops, including *Brassica* vegetables (Table 1). DM *R* genes or loci such as BraRHP1Q (syntenic with *A. thaliana* chromosome 3), BraDM, and Bra-DM04 have been mapped on chromosome A01, A08, and A04, respectively, in *B. rapa* vegetables (Figure 3, Table 4) [173]. In Chinese cabbage, development-stage-specific DM-resistant QTL (seedling: sBrDM8, young plant: yBrDM8; rosette: rBrDM8, and heading hBrDM8) are identical to the BraDM locus [174]. BraDM linked InDel (Brb062-Indel_230_), CAPS (Brb094-DraI_787_, Brb094-AatII_666_, and Brb043-BglII_715_), SNP (Brh019-SNP_137_), and SSR (bru1209, homologous to KBrB058M10) markers showed 58.3–74.2% accuracy in selecting DW-resistant lines from the DH population (Table 4) [175]. A SCAR marker, SCK14-825, developed from K14-1030 identified a sequence homology to a sequence of bacterial artificial chromosome (BAC) clones (Table 4) [176]. Two SSR markers (kbrb058m10-1 and kbrb006c05-2) were designed from homologous BAC sequences and mapped to the BrDM QTL interval [176]. K14-1030, kbrb058m10-1, and kbrb006c05-2 markers showed high selection accuracy in MAS for DM resistance breeding in *B. rapa* ssp. *pekinensis* [176]. The sBrDM8 locus has a candidate gene, Bra016457, which encodes a serine/threonine kinase family protein [174]. Fine mapping of the BraDM locus identified three protein-coding wall-associated kinase (WAK) family genes [Bra016426 (*BrWAK1*), Bra016427 (*BrWAK2*), and Bra016428 (*BrWAK3*)] [177]. Overexpression of *BrWAK1* in a susceptible line significantly increased resistance against the downy mildew pathogen. The defense response was triggered by downstream regional MAPK activation, and expression of *BrWAK1* causes an interaction between brassinosteroid insensitive 1 associated kinase (*BrBAK1*) and MAPK, resulting in a significantly increased DM resistance in Chinese cabbage [177]. The *BrRLP47* (Bra032746), *BrRLP48* (Bra032747), *BrLRR1* (Bra032740), and *BrLRR2* (Bra032741) genes were identified as DM *R* genes within the Bra-DM04 QTL in *B. rapa* [178]. Overexpression of *BrRLP47* and *BrRLP48* enhanced DM resistance in a susceptible line. The promoter of *BrRLP48* in resistant lines contains SA- and JA-responsive transcriptional elements, whereas such elements are absent from susceptible lines. Thus, DM inoculation or SA treatment significantly induced expression of *BrRLP48* in the resistant line, making it a strong candidate for regulating DM resistance in *B. rapa* [178]. *BoDMR2*, *Pp523*, and *Ppa3* genes for DW resistance have been mapped in *B. oleracea* (Figure 4, Table 4). *BoDMR2* was mapped to a 300 kb interval on chromosome C07 at the adult stage in cabbage [179]. The candidate Bo7g117810 gene in the BoDMR2 locus exhibited a conserved 3 bp insertion in the susceptible line and showed 2.5-fold lower expression than in the resistant line. The InDel marker based on Bo7g117810 can be used for accurate selection of DM-resistant cabbage varieties [179]. In the locus covering the *Pp^523^* gene on chromosome C08 of broccoli, two of the three SCAR markers (SCJ19, and SCAFB1), which have polymorphic restriction sites, function as co-dominant CAPS markers. These markers are useful for MAS in breeding programs (Table 4) [180]. The OPK17_980 and SCAFB1 markers of the locus covering the *Pp^523^* gene of *B. oleracea* correspond to synthetic regions of At1g01220 and At1g07420 in *A. thaliana*, respectively [181]. A QTL, PpALG1, found in cotyledon and adult plants of *B. oleracea* var. *tronchuda* was located in a genomic region similar to the region covering *Pp^523^* gene on chromosome C08 [182]. The *Ppa3* (a single dominant locus) and *Ppa^207^* genes have been mapped to chromosome C02 in cauliflower (Table 4). The *Ppa3* gene was used for pyramiding with ScOPO-04_833_ in “Pusa Meghna” cauliflower together with the black rot-resistant gene (*Xca1bo*) [183], and pyramided lines showed resistance against both pathogens.

Gene expression profiles of resistant and susceptible lines of Chinese cabbage and pak choi following *H. brassicae* infection have shown a predominant role of the SA signaling pathway in DM resistance [184,185]. *PAL1*, *ICS1*, *NPR1*, *PR1*, *PR5*, *WRKY70*, *WRKY33*, *CML43*, *CNGC9*, and *CDPK15* genes are involved in the DM resistance mechanisms of *B. rapa* [185]. Genome-wide expression analysis using resistant and susceptible lines of Chinese cabbage showed upregulation of Bra010447 (*PR-1*; pathogenesis-related), *PHENYLALANINE AMMONIALYASE* (*PAL*; Bra029831 and Bra005221), and glutaredoxin family protein (*GRX*; Bra030102 and Bra002306) genes in the resistant lines, indicating their functional roles in resistance mechanisms against DM [186]. The involvement of two *H. parasitica* induced genes, *Bcchi* and *BcAF*, was identified in the response to DM infection in non-heading Chinese cabbage [187]. A comparative transcriptomic analysis between the resistant line ‘Suzhou Qing’ and the susceptible line ‘Aijiao Huang’ of non-heading Chinese cabbage identified four differentially expressed transcript-derived fragments (TDFs). This study revealed 25.3-, 25.1-, 100-, and 15.8-fold increases in the expression of TDF14 (*BcLIK1*_A01), TDF42 (*BcCAT3*_A07), TDF75 (*BcAAE3*_A06), and TDF88 (*BcAMT2*_A05), respectively, in the resistant line at 24 or 48 h post-inoculation (hpi) [188]. Higher transcription levels of these TDFs might be associated with a DM resistance mechanism in non-heading Chinese cabbage. Non-coding RNAs are also involved in DM resistance of *Brassica* vegetables. Silencing a natural antisense transcript (NAT, *MSTRG.19915*), which overlaps with *BrMAPK15*, increased DM resistance of Chinese cabbage [189]. The resistance hotspot on chromosomes A08 and C08 in *B. rapa* and *B. oleracea*, respectively, controls the DM defense system through downstream activation of the SA signaling pathway. By decreasing race-specificity, molecular markers can accelerate breeding and develop durable DM resistance varieties of *Brassica* vegetables.

**Table 4:** Downy mildew-resistant loci identified in *Brassica* vegetables.

Parents	Population	Marker System	Major Loci	Chr	Linked/Flanking Markers (PL in Mb) ^1^	Reference
***B. rapa***						
Chinese cabbage; RS1 (R), SS1 (S)	F_2_, F_3_, F_4_, BC1	RAPD, SCAR	BrRHP1	A01	BrPERK15A	[173]
Chinese cabbage; 91–112 (S) and T12–19 (R)	DH, BC_2_	SNP, SLAF	BraDM	A08	PGM~K14-1030	[174]
	DH	RAPD	BraDM	A08	K14-1030~KBrB058M10	[175]
	DH	SSR	BraDM	A08	K14-1030~kbrb006c05-2	[176]
	DH, BC_1_, BC_2_, BC_3_	SNP	BraDM	A08	A08-17629022~SNP-428-2FF	[177]
BY (*B. rapa* ssp. *pekinensis*) MM (*B. rapa* ssp. *rapifera*)	DH, F_2_	SNP	Bra-DM04	A04	A04_5235282 and A04_5398232	[178]
***B. oleracea***						
Pusa Himjyoti (S) and BR-2 (R)	F_2_	RAPD, ISSR, SSR	*Ppa3*	-	OPC14_1186_~OPE14_1881_	[190]
R pool, S pool	-	InDel	*BoDMR2*	C07	W8-3 (46.8)~W7-22 (47.2)	[179]
Broccoli; GK97362 (S), OL87125 (R)	F_2_	RAPD, AFLP, SSR, ISSR	*Pp523*	C08	OPK17_980~AT.CTA_133/134	[191]
	F_2_	RAPD, AFLP, SCAR, CAPS	*Pp523*	C08	SCR15~SCAFB1	[180]
Broccoli; OL 87098 (S), OL87125 (R)	F_2_	RAPD, SSR, ISSR, AFLP, SCAR, BAC-end derived STS	*Pp523*	C08	AAG.CTA_113y~AAG.CTA_1200	[192]
	F_2_	BAC-end derived STS	*Pp523*	C08	67---167K22_F_cod~AAC.CAA_1200	[181]
*B. oleracea* var. *tronchuda* Bailey R and S lines	F_2_, F_3_	RAPD, ISSR, SSR, BAC-end derived markers	PpALG1	C08	31N6_Ry~CB10045A	[182]
Cauliflower; BR-2 (R), and Pusa Himjyoti (S)	F_2_	RAPD, ISSR	*Ppa3*	C02	OPC14_1186_~OPE14_1881_	[193]
Cauliflower; Pusa Sharad (S), DMR-2-0-7 (R)	RIL	SSR	*Ppa^207^*	C03	BoGMS0486 (2.9) and BoGMS0900 (23.2)	[194]

^1^ PL represents the physical location in megabase (Mb) that has been mentioned in the parenthesis in the linked marker column. S—susceptible; R—resistant; DH—double haploid; F_2_, F_3_, and F_4_—second, third, and fourth filial generations; BC_1_, BC_2_, and BC_3_—backcrossed first, second, and third generation; RIL—recombinant inbred line; RAPD—random amplified polymorphic DNA; SCAR—sequence-characterized amplified region; SNP—single-nucleotide polymorphism; SLAF—specific-locus amplified fragment; SSR—simple sequence repeat; ISSR—inter-simple sequence repeat; InDel—insertion–deletion; AFLP—amplified fragment length polymorphism; CAPS—cleaved amplified polymorphic sequences; BAC—bacterial artificial chromosome; STS—sequence-tagged site.

### 3.4. Black Rot

QTLs for resistance against the bacterial black rot pathogen, *Xanthomonas campestris* pv. *campestris* (Pam.) Dowson (*Xcc*), have been identified in various *Brassica* vegetables (Figure 3 and Figure 4, Table 5). Although many QTLs have been reported, no *R* gene has yet been conclusively identified. Currently, there is a strong need for the development of DNA markers for *Xcc* resistance, due to both limited availability of resistance resources and the pressing demand for breeding resistant cabbage varieties. Two closely linked resistance loci against *Xcc* races 1 and 4 were detected on chromosome A06 in *B. rapa* [195]. A major QTL, Xca1bo, was identified on chromosome C02 in cauliflower [196]. A major QTL on chromosome C02 (QTL-1) of *B. oleracea*, along with its syntenic region in *A. thaliana* (A05: 5.3–7.4 Mb), was enriched with TIR-NBS-LRR family genes [197]. One major QTL [XccBo(Reiho)2 on chromosome C08] and two minor QTLs [XccBo(Reiho)1 on chromosome C05 and XccBo(GC)1 on chromosome C09] for *Xcc* resistance were identified in *B. oleracea* [198]. The XccBo(GC)1 QTL overlaps with a QTL from another study [199], and XccBo(Reiho)1 QTL overlaps with QTL-3 [197]. Therefore, consistency of these two QTLs, XccBo(GC)1 and XccBo(Reiho)1, contribute to *Xcc* resistance in *B. oleracea*. Two major QTLs were identified on chromosome C01 of cabbage across repeated trials within the physical position 14,884,502–16,579,946 bp (BRQTL-C1_1) and 18,227,386–37,119,290 bp (BRQTL-C1_2) [200]. NBS-LRR encoding candidate genes (Bo1g094680 and Bo1G094710) were identified from BRQTL-C1, which correspond to syntenic genes in *B. rapa* (A01: Bra031456 and Bra031455, respectively) and *A. thaliana* (AT1G61100 and AT1G61105, respectively) [200]. Four QTLs for *Xcc* resistance in *B. oleracea* (Xcc1.1, Xcc6.1, Xcc8.1, and Xcc9.1) were identified with Xcc9.1 being novel [201]. Of these four QTLs, Xcc1.1 corresponds to BRQTL-C1_1 and BRQTL-C1_2 on chromosome C01, Xcc6.1 corresponds to a minor QTL BRQTL-C6 on chromosome C06, and Xcc8.1 corresponds to XccBo(Reiho)2 on chromosome C08 [200,201]. An overlapping resistance QTL for *Xcc*, qCaBR1, was detected across two seasons within the 29,853,043–34,373,426 bp region on chromosome C06 [202]. Four potential candidate genes (Bo6g098480, Bo6g099850, Bo6g101010, and Bo6g106440) within this interval showed higher expression in resistant lines than in susceptible lines at different time points following *Xcc* inoculation, with expression patterns similar to the *PR1* gene [202]. Another candidate *R* gene, Bol031422, for *Xcc* resistance was found on chromosome C08, which has a 3 bp insertion/deletion; a marker linked to the Bol031422 gene (BR6-InDel) can be used to detect variations in *Xcc* resistance against races 6 and 7 [203].

**Table 5:** Black rot-resistant loci identified in *Brassica* vegetables.

Parents	Population	Race	Marker System	Major Loci	Chr/LG	Linked/Flanking Markers	Reference
***B. oleracea***							
Cabbage BI-16 (R), Broccoli OSU Cr-7 (S)	F_3_	-	RFLP	QTL-LG1, QTL-LG9	C01 (LG1), C09 (LG9)	wg6g5, wg8a9b	[199]
Cabbage: January King (R) × Golden Acre (S)	F_2_	-	RAPD	Xcc *R* gene	-	C-11_1000_	[204]
Broccoli GC P09 (S), Cabbage Reiho P01 (R)	F_3_	1	SRAP, CAPS	2 loci	LG2	CAM1~GSA1	[205]
					LG9	F12-R12-e~BORED	
Cabbage CY (R), Broccoli BB (S)	F_3_	1	EST-SNP	QTL-1 *	C02	BoCL5989s~BoCL5545s	[197]
				QTL-2	C04	BoCL1384s~BoCL7837s	
				**QTL-3**	C05	BoCL5860s~BoCL4231s	
Broccoli GC P09 (S), Cabbage Reiho P01 (R)	F_2_, F_3_	1	CAPS, SSR, SNP	**XccBo(Reiho)2**	C08	BoGMS0971, OL12D05	[198]
				**XccBo(Reiho)1**	C05	BoGMS1330,	
				XccBo(GC)1	C09	CB10509, CB10459, pW143	
Cauliflower	F_2_	2	RAPD, ISSR, SSR	Xca1bo	C03	RAPD_04833_~ISSR_11635_	[206]
Cabbage		-	SNP and EST based dCAPS, MIP, IBP, SSR, InDel	BRQTL-C1_1 *	C01	BnGMS301, BoESSR726, BoESSR145 (14.8~16.5)	[200]
				BRQTL-C1_2 *	C01	BoESSR089, BoEdcaps4, BnGMS299 (18.2~37.1)	
				BRQTL-C3	C03	B041F06-2 (19.7~22.8)	
				**BRQTL-C6**	C06	Ol10-G06 (7.4~10.4)	
Cauliflower	F_3_	1	RAPD, ISSR, SCAR	Xca1bo	C03	ScOPO-04833 and ScPKPS-11635	[196]
Cabbage, inbreed lines	-	1-7	SSR, InDel	-	C01	BnGMS301-BoESSR726	[207]
					C03	BoESSR291	
					C06	OI10G06	
					C08	BoGMS0971	
Broccoli ‘Early Big’ (S), Chinese kale ‘TO1000DH3’ (R)	DH	1		**Xcc1.1**, **Xcc6.1**, **Xcc8.1**, Xcc9.1	C01, C06, C08, C09	C01: BRQTL-C1_1, BRQTL-C1_2 (1), C06: BRQTL-C6 (1), C08: XccBo(Reiho)2 (2)	[201]
Cabbage	F_2_, F_3_	1	GBS, SNP	qCaBR1	C06	-(29.8~34.3)	[202]
***B. rapa***							
Turnip (S), Pak choi (R),	F_2_	4	RAPD	-	-	WE22, WE49	[208]
R-o-18 Yellow–Sarson (S), B162 (R),	F_2_	1	AFLP	XccR1d-1 *	A06	E11M50_280b	[195]
		4		XccR1d-1 *	A06	E12M61_215b	
		1		XccR4i-1 *	A06	E12M48_171r	
		4		XccR4i-1 *	A06	E12M61_215b	
		1, 4		XccR4i-2	A02	E11M59_178r	
				XccR4i-3	A09	E12M48_1>330b	
P115 × P143	DH	1, 3, 4, 6	RAPD, RFLP, AFLP	19 QTLs	A01-A07, A09	Many	[209]
P175 × P143	DH			13 QTLs	A01-A06, A08, A10	Many	
Radish	F_2_, F_3_	-	RAD-seq, SNP, InDel	qBRR2	LG2	-	[210]
				qBRR7	LG7		

Similar-colored bold QTL represents the QTL in similar region and * represents over detected QTL. S—susceptible; R—resistant; DH—double haploid; F2 and F3—second and third filial generations; LG—linkage group; RFLP—restriction fragment length polymorphism; RAPD—random amplified polymorphic DNA; SRAP —sequence-related amplified polymorphism; CAPS—cleaved amplified polymorphic sequences; dCAPS—derived CAPS; EST—expressed sequence tag; SNP—single-nucleotide polymorphism, SSR—simple sequence repeat, ISSR—inter-simple sequence repeat; InDel—insertion–deletion; MIP—MITE insertion polymorphism; IBP —Intron-based polymorphic; SCAR—sequence-characterized amplified region; GBS—genotyping-by-sequencing; AFLP—amplified fragment length polymorphism; RFLP—restriction fragment length polymorphism; RAD-seq—Restriction-site Associated DNA Sequencing.

### 3.5. Turnip mosaic virus (TuMV)

TuMV, genus Potyvirus, family Potyviridae, is causing major viral diseases affecting *Brassica* vegetables with significant yield losses (Table 1). Over 20 resistance genes/loci against TuMV have been identified. TuMV resistance-associated QTLs have been mapped across chromosomes A03, A04, A05, A06, A07, and A10 [211] (Figure 3, Table 6). Moreover, TuMV *R* genes such as *ConTR01*, *retr01*, *retr02*, *Rnt1*, *TuRBCH01*, *TuRB07*, *TuRB01b*, and *TuRBCS01* have been mapped in *B. rapa* (Figure 3, Table 6). A single dominant gene, *ConTR01* (located on the upper arm of chromosome A08), is epistatic to a single recessive gene, TuMV resistance 01 (*retr01*), located on the upper arm of chromosome A04 in Chinese cabbage [211]. Both of these genes coincide with a region encoding the eIF(iso)4E protein in the A subgenome of *B. napus*, and likely in *B. rapa* as well [212]. The *retr02* gene (Bra035393) encodes an eIF(iso)4E protein and is a candidate *R* gene for TuMV resistance [213]. Bra035393 contains an A/G polymorphism in exon 3 between resistant and susceptible lines [213]. Gene editing of eIF(iso)4E (Bra035393) using CRISPR/Cas9 technology has been shown to confer resistance against TuMV [214]. A TuMV resistance locus on chromosome A06 has been consistently detected by different research groups (Table 6). Chromosome A06 has a major TuMV resistance locus covering a Bra018863, which encodes a functional CC-NBS-LRR protein [215]. Genetic analysis has identified BraA06g035130.3C, encoding a CC-NBS-LRR protein, as a candidate for dominant *R*-mediated resistance gene on chromosome A06 [216].

**Table 6:** Turnip mosaic virus (TuMV) disease resistance loci in *B. rapa* vegetables.

Parents/F1	Population	Isolate/Race	Marker System	Major Loci/R-Gene (Chromosome)	Linked/Flanking Markers	Reference
Chinese cabbage; BP079 (R) and RLR22 (S)	BC_1_, BC_1_S_1_	CDN1, CZE1	RFLP	*retr01* * (A04)	pN202e1	[217]
				*ConTR01* * *(A08)*	pO85e1	
Chinese cabbage; 91-112 (R) and T12-19 (S)	DH	C4	AFLP, RAPD, SSR, SCAR	Tu1 (A05)	A04-850~CA_TG470	[218]
				Tu2 (A10)	X12-850	
				Tu3 (A03)	U10-1500~CA_TC157	
				Tu4 (A04)	CT_TC710	
Chinese cabbage; A52-2 (R) and GCⅣ (S)	F_2_	C3	AFLP, EST-PCR-RFLP	TuR1 (A03)	E41M5808~E39/M5305	[219]
				TuR2 (A03)	E39/ M505~E42/M5710	
				TuR3 (A07)	E38/ M5401~E38/M5106	
				TuR4 (A07)	E38/M5106~HpaII650	
Chinese cabbage; Y195-93 (R) and Y177-12 (S)	DH	C4	-	Tu1 (A03)	E36M47-7	[220]
				Tu2 (A04)	E33M60-5	
				Tu3 (A06)	E36M59-5	
Pak choi; Q048 (R) and A168-5D (S)	F_2_	C5	AFLP	*TuRBCH01* *	EaccMctt3~EaccMctt1	[221]
Pak choi	F_2_	C5	AFLP, SSR	*TuRBCH01* * (A06)	E36M62-3~E44M48-1	[222]
Chinese cabbage; 73 (R) and 71-36-2 (S)	F_2_	C4	EST-SSR	*retr02* *	HCC259	[223]
Chinese cabbage; AS9 (R) and SS11 (S)	F_2_	1	InDel	*Rnt1* * (A06)	BRMS-221~BRMS-223	[224]
Chinese cabbage; BP8407 (S) and Ji Zao (R)	F_2_	C4	SSR, InDel	*retr01* * (A04)	pN202e1 (*retr01*)	[213]
				*retr02* * (A04)	BrID10694~BrID101309, and Scaffold000060/Scaffold000104	
Chinese cabbage; GJS2A (S) × SB18 (R) and SB22 (R) × SB24 (S)	F_2_	C3	SNPs, SCAR	*trs* *^a^ (A04)	Scaffold000104~Scaffold040552	[225]
*B. rapa*; VC1 (R) and SR5 (S)	DH, F_2_, BC_1_	C4	SSR	*TuRB07* * (A06)	H132A24-s1~KS10960	[215]
Chinese cabbage	BC_1_	-	RFLP	*TuRB01b* * (A06)	pN101e1~pW137e1	[226]
*B. rapa*; VC40 (R) and SR5 (S)	DH	C4	SNPs, InDel, SSR	TuMV-R (A06)	No343~CUK_0040i	[227]
Chinese cabbage; 43 P1 (R), 88 P2 (S)	F_2_, BC_1_	C4	SSR, InDel, EST	*TuRBCS01* * (A04)	BrID10723~SAAS_mBr4055_194	[228]
Chinese cabbage	BC_1_	-	SSR, SSP	*TuRBCS01* * (A04)	SAAS_mBr4072_240~Bra025493-1	[229]
*B. rapa*; B80124 (R), B80450 (S)	F_2_	C4	SNPs, KASP	qtl (A06)	A06S11–A06S14	[216]
*B. rapa* ssp. *rapa*; BR05058 (R), S22561 (S)	BC_1_	CDN1, GBR6	SNP	QTL (A06)	A06-p49446208~A06-p50287184	[230]

* represents gene; ^a^ represents tightly linked to *retr02*; S—susceptible; R—resistant; DH—double haploid; F_2_—second filial generations; BC_1_—backcrossed first generation; BC_1_S_1_—backcross segregating first generation; SNP—single-nucleotide polymorphism; RAPD—random amplified polymorphic DNA; SSR—simple sequence repeat; SCAR—sequence-characterized amplified region; AFLP—amplified fragment length polymorphism; InDel—insertion–deletion; EST—expressed sequence tag; CAPS—cleaved amplified polymorphic sequence, indel, RFLP—restriction fragment length polymorphism; *retr01*—recessive TuMV resistance 01 (a recessive single gene); *ConTR01*—conditional TuMV resistance 01 (a dominant single gene); *TuRBCH01*—a TuMV-C5 resistance gene; *Rnt1*—a TuMV resistance gene population.

### 3.6. Sclerotinia Rot, Soft Rot, Alternaria Leaf Spot, Blackleg, and White Rust Diseases in Brassica Vegetables

Sclerotinia rot or stalk rot (SR) caused by the necrotrophic fungus *Sclerotinia sclerotiorum* is less aggressive on *Brassica* vegetables than oilseed rape (*B. napus*). Therefore, genetic studies such as the identification of resistance QTL for SR resistance have been less frequently conducted in *Brassica* vegetables (Table 7). Leaf- and stem-resistance QTLs have been co-localized between the SWUC663 and SWUC731 markers on chromosome C09 of *B. oleracea*, a region syntenic to the region from 1.6 to 4.3 Mb on chromosome A09 of *B. rapa*. This region contains genes encoding LRR, CC-NBS-LRR, and zinc finger family proteins [231]. The SR QTL region on chromosome C09, which includes Bo7g104800, overlaps with the YR gene [232]. Introgression of this resistance locus from chromosome C09 into *B. rapa* using DNA markers resulted in a 1.4- and 1.7-fold increase in sclerotinia leaf- and stem-rot resistance, respectively [233]. These findings suggest the QTL on chromosome C09 has potential for developing SR-resistant *Brassica* vegetable varieties.

*Pectobacterium carotovorum* subsp. *carotovorum* (*Pcc*) causes soft rot in *Brassica* vegetables. In *B. rapa*, *UDP-glucose 4-epimerase1* (*BrUGE1*), *BrUGE4*, and *WRKY7* genes were induced following *Pcc* inoculation, suggesting their involvement in resistance mechanisms [234]. In *B. rapa*, three QTLs associated with *Pcc* resistance have been identified on chromosomes A02 and A07, and six genes in two QTLs on chromosome A07 were identified as candidates for *Pcc* resistance [235] (Table 7). It suggests that chromosome A07 may have a potential role in resistance against *Pcc*. However, according to our knowledge, there is no definitive study to identify genomic regions for *Pcc* resistance in *B. oleracea*.

Two *Alternaria* species (*A. brassicicola* and *A. brassicae*) invade *Brassica* vegetables and cause Alternaria leaf spot disease (Table 1). A major QTL governing resistance against *A. brassicae* was detected in *A. thaliana* [236]. In the *A. thaliana–A. brassicae* pathosystem, three *R* genes against *A. brassicae* (At1g06990, At3g25180, and At5g37500) were identified [237]. An 1-amino-cyclopropane-1-carboxylic acid oxidase (ACCox1), a putative leucine-rich serine-threonine kinase, a polygalacturonase inhibitor protein (PGIP), and a WRKY TF were identified as contributors in the host plant defense response during the interaction between *A. brassicicola* and *B. oleracea* [238]. The resistance mechanism against *A. brassicicola* in *Brassica* vegetables is triggered through biosynthesis of 4-methoxy indole-3-ylmethyl glucosinolate (4OH-I3M or 4-methoxyglucobrassicin), which is regulated by WRKY33 [239]. WRKY33 activates CYP81F2, IGMT1, and IGMT2 to convert indole-3-ylmethyl glucosinolate (I3G) to 4MI3G in *A. thaliana* and Chinese kale [239]. QTLs responsible for *A. brassicae* resistance have not been identified in the diploid genome of *B. rapa* (A genome) and *B. oleracea* (C genome).

Blackleg is caused by the fungal pathogen *Leptosphaeria maculans* (Desm.) Ces. and de Not; this disease is a serious threat to canola as well as cabbage (Table 1). A blackleg-resistant QTL, which contains six *R* genes, was identified in a 160 kb region on chromosome A06 of Chinese cabbage [240]. The blackleg resistance locus, *LepR1*, in *B. napus*, is syntenic to chromosome C02 of *B. oleracea* covering genes encoding NBS, LRR, TIR, F-box, and RLK domains. *LepR4* from *B. napus* is collinear with the 9.07–14.85 Mb region on chromosome A06 of *B. rapa*, which harbors several NBS-LRR encoding genes (Bra018037, Bra018057, Bra018198, and Bra019483) [241]. Another NBS-LRR encoding gene, Bo2g131620, had higher expression levels in resistant lines, suggesting its potential role in resistance mechanisms in cabbage [242,243]. Bol033373 and Bol026044 may be involved in defense mechanisms against blackleg disease of cabbage [244]. The gene product of *Rlm1*, a major *R* gene, located on chromosome A07 of *B. napus* interacts with the *L. maculans* effector protein AvrLm1, resulting in an effector-triggered defense (ETD) response [37,53]. A homolog of the *Rlm1* gene was identified on chromosome C06 of cabbage where a TIR-NBS family gene (Bol040038) was upregulated, and three genes were differentially expressed in resistant lines [245]. Chromosome A06 and C06 might contain potential *R* gene against *L. maculans* in *B. rapa and B. oleracea*, respectively.

White rust is caused by an obligate biotrophic oomycete pathogen, *Albugo candida*. *ALPHA CARBONIC ANHYDRASE* 1 (*ACA1*), a resistance gene against *A. candida* race 2 and *PUB1* (leaf pubescence loci) were mapped on chromosome A04 of *B. rapa* (Table 7) [246]. DEGs between *A. candida*-resistant and -susceptible komatsuna varieties have been identified. Genes involved in SAR, regulation of defense response, and programmed cell death were upregulated in the resistant variety [45]. *A. candida* inoculation changed expression levels of SA responsive genes in both resistant and susceptible varieties, but different sets of genes were affected in each variety [45]. *A. candida* inoculation was shown to activate SAR, immunity, and defense response, suggesting that SAR was involved in downstream of the ETI signaling pathway [247].

**Table 7:** Sclerotinia rot, soft rot and white rust resistant loci identified in *Brassica* vegetables.

Parents	Population	Race	Marker System	Major Loci	Chr	Linked/Flanking Markers (Physical Position in Mb)	Reference
**Sclerotinia rot (*Sclerotinia sclerotiorum*)**							
*B. incana* ‘C01’ (R), *B. oleracea* var. *alboglabra* ‘C41’ (S)	F_2_	-	SSR, AFLP, SRAP	qLR	C01	SWUC59/170~Na12-C08	[231]
				qLR-5	C09	SWUC679~SWUC635	
				qLR-6	C09	SWUC700~SWUC711	
				qSR-1	C09	SWUC611~Ra2-F11	
				qSR-2	C09	SWUC700~SWUC711	
*B. villosa* ‘BRA1896’ (R), *B. oleracea* ‘BRA1909’ (S)	F_2_	-	SNP	pQTLa	C01	Bn-scaff_15747_1-p105633~Bn-scaff_22790_1-p1026422 (14.2~17.4)	[248]
				*pQTLb1*	C03	Bn-scaff_16614_1-p734250~Bn-scaff_16614_1-p174856 (2.0~3.1)	
				*pQTLb2*	C07	Bn-scaff_16069_1-p2611780~Bn-scaff_16069_1-p4306874 (42.3~44.0)	
				*lQTLb*	C07	Bn-scaff_16110_1-p975852~Bn-scaff_16110_1-p426547 (47.3~47.9)	
**Soft rot (*Pectobacterium carotovorum* or *Erwinia carotovorum*)**							
Chinese cabbage A03 (S), pakchoi ‘Huaguan’ (R)	F_2_	-	SNP	DRQTL-1	A02	A02-668352~A02-761454	[235]
				DRQTL-2	A02	A02-4366585~A02-5305993	
				DRQTL-3	A07	A07-26520444~A07-26625030	
**White rust (** ***Albugo candida*** **)**							
*B. rapa*	F_2_, F_3_	2, 7	RFLP	*ACA1*, *PUB* genes	A04	ec2b3a~wg6c1a	[246]
*B. rapa* ssp. *oleifera*; Bor4206 (S), Bor4109 (R)	F_2_	7a, 7v	RAPD, AFLP	*-*	A02	*Z19a*	[249]

S—susceptible; R—resistant; F_2_ and F_3_—Second and third filial generations; SSR—simple sequence repeat; AFLP—amplified fragment length polymorphism; SRAP—sequence-related amplified polymorphism*;* SNP—single-nucleotide polymorphism; RFLP—restriction fragment length polymorphism; RAPD—random amplified polymorphic DNA.

## 4. Hostplant Epigenetic Resistance Mechanisms

### 4.1. Epigenome Analysis and Epigenomic Defense Response in Brassica Vegetables

Epigenetic regulators play a crucial role in transcriptional regulation in *Brassica* vegetables. DNA methylation, histone modifications, and chromatin remodeling are the most common epigenetic mechanisms [26,250,251]. DNA methylation refers to the addition of a methyl group (CH_3_) to cytosine bases in DNA, forming 5-methylcytosine (5mC) [26]. In plants, DNA methylation can occur in sequence contexts: CG, CHG, and CHH (where H represents any base pair except G) [26]. In plants, DNA is wrapped around histone octamers each composed of two copies of the core histone proteins H2A, H2B, H3, and H4. Post-transcriptional modifications (PTMs) of histone tails, such as methylation (me), acetylation (ac), phosphorylation (ph), and ubiquitination (ub) serve as epigenetic marks [26]. Acetylation of histone H3 (H3ac), H4ac, trimethylation of histone H3 at lysine 4 (H3K4me3), H3K36me3, and monoubiquitination of H2B (H2Bub1) are generally associated with transcriptional activation, whereas histone deacetylation, H3K9me2, H3K27me3, and H2Aub1 are associated with transcriptional repression. Transcriptional reprogramming via DNA methylation or PTMs plays a central role in the regulation of plant defense mechanisms [252].

Epigenetic studies beyond stress responses, especially DNA methylation and histone methylation (H3K4me3, H3K9me2, H3K27me3, and H3K36me3), have significantly advanced our understanding of transcriptional regulatory mechanisms underlying plant development and gene expression in *Brassica* vegetables. The whole genome bisulfite sequencing (WGBS) of a Chinese cabbage inbred line revealed that genome-wide CG sites (36.5%) were highly methylated compared to CHG (13.4%) and CHH (5.3%) sites. Similar DNA methylation patterns (CG—73.7%, CHG—33.8%, CHH—13.0%) were observed in interspersed repeat regions (IRRs) [253]. In a semi-winter type *B. rapa* var. *oleifera*, the higher genome-wide DNA methylation levels were also observed in CG sites (52.4%), followed by 31.8% in CHG and 8.3% in CHH sites using the reduced representation bisulfite sequencing (RRBS) method [254]. *B. rapa* has single/double/triple copies of genes due to whole genome triplication. DNA methylation levels in single copy genes were higher than in multiple copy genes, and transcription levels were positively (or negatively) associated with DNA methylation levels, suggesting the potential role in polyploid genome evolution in *Brassica* vegetables [254,255]. There is no correlation between DNA methylation and gene expression, but DNA methylation plays a role in the functional diversification of duplicated genes [256]. In contrast, DNA methylation is closely related to silencing transposable elements (TEs) in both *B. rapa* and *B. oleracea* species; TEs were highly methylated in both species, although the distribution and levels of methylation differed between species [255]. Genes with DNA methylation in introns, as well as in 200 bp up- and downstream of gene bodies, exhibited reduced expression levels in *B. rapa* inbred lines [257]. There was a non-linear relationship between CG gene body methylation and gene expression levels, for example, moderate levels of CG methylation in gene body are associated with a high level of gene expression [256]. Transcriptional changes by DNA methylation are associated with overwintering memory [258], male germline and pollen development [259,260], inbreeding depression in heading traits [261], yield heterosis [262], and responses to biotic and abiotic stresses [263,264] in *Brassica* vegetables.

The chromatin remodeling factor *BrCHR39*, an apical dominance regulating gene of SNF2—sucrose non-fermenting2, histone linker, PHD—plant homeodomain, RING—really interesting new gene, and helicase (SHPRH) subfamily, was silenced using RNA interference (RNAi) to compare genome-wide DNA methylation with wild-type [265]. In *BrCHR39*-silenced plants, differentially methylated genes (DMGs) in the auxin-related pathway such as *AUX1*, *AAO1*, *IAA*, *ARF1/3*, *SAUR15/72*, and *GH3* were hypermethylated in stems with lower gene expression, while auxin- and cytokinin-related genes such as *ARF8/9*, *SAUR32/41*, *CKI1*, and *ARR7/9* were hypomethylated in the bud, resulting in higher expression levels [265]. These findings suggest that chromatin remodeling can also modulate DNA methylation to regulate gene expression in *B. rapa*.

The gene regulatory mechanisms of H3K4me3 (activating), H3K9me2 (repressing), H3K27me3 (repressing), and H3K36me3 (activating) are conserved across *Brassicaceae* species and other eukaryotes. About one-third of all protein-coding genes were marked by H3K27me3, a modification correlated with lower levels of transcription in *B. rapa* var. yellow sarson (*ssp. trilocularis*). Reduced levels of H3K27me3 at the AGAMOUS-like genomic region were associated with increased expression of genes located in that region in *braA.clf-1* mutants (deficient in CURLY LEAF, a polycomb repressor complex 2 component) [266]. In *B. rapa* inbred lines, H3K4me3, H3K36me3, and H3K27me3 marks were observed in 16,759, 11,844, and 10,456 genes, respectively [267,268]. Bivalent histone modifications, a simultaneous presence of active H3K4me3 and repressive H3K27me3 marks on the same genomic regions, were observed in 35.4% of the genes in *B. rapa* [268]. Although these bivalently marked genes exhibit high tissue specificity, their expression levels were comparable to those of H3K27me3 marked genes. These bivalently histone methylated genes encode important TFs such as *LFY*, *WRKY*, *ERF*, and *IAA* [268]. However, genes marked with both H3K36me3 and H3K27me3 showed expression levels similar to those marked by H3K4me3 with less tissue specificity [268]. Functional associations among the histone modifications and DNA methylation have also been examined. H3K9me2 showed a positive correlation with DNA methylation, whereas H3K4me3, H3K27me3, and H3K36me3 were negatively associated with DNA methylation [253,267,268,269].

In addition, the relationship between long non-coding RNAs (lncRNAs)—including long intergenic non-coding RNAs (lincRNAs), intronic non-coding RNAs (incRNAs), and natural antisense transcripts (NATs)—and epigenetic marks is an emerging area of study in *Brassica* vegetables. Overlaps between lncRNAs and regions marked by DNA methylation or histone modifications suggest potential roles in transcriptional regulation [270,271,272]. Studies continue to explore how lncRNAs may influence gene expression through interactions with histone modifications and DNA methylation landscapes.

### 4.2. Lessons from Arabidopsis for Shaping the Epigenetic Landscape in Defense Response

QTLs have been identified in *Brassica* vegetables for resistance against various pathogens (Table 2, Table 3, Table 4, Table 5, Table 6 and Table 7). Epialleles are genetically identical but epigenetically distinct individuals that can stably inherit their characteristics across generations and play a crucial role in resistance against biotic stress by altering transcriptional activity [273,274]. Recent advancements in molecular research highlight the need to progress these further by identifying epigenetic QTL (epiQTL). These epiQTLs could link resistance genes that are regulated epigenetically, a concept already explored in *A. thaliana* using epigenetic recombinant inbred lines (epiRILs). EpiRILs are similar to conventional RILs, but they are genetically uniform and differ in their DNA methylation profiles [275]. The use of an epiRIL population for trait mapping is known as epigenome mapping, and the identified QTLs are referred to as epiQTLs [276,277,278]. In *A. thaliana*, 20 epiQTLs for CR have been identified, and 6 of them co-localized with previously known *CR* genes or QTLs [279]. More recently, 31 epiQTLs for CR have been identified, and 21 of them are also involved in resistance against heat, drought, and flooding [280].

Mutants with hypomethylation (e.g., *nrpe1*) show resistance against the downy mildew (DM) pathogens in *A. thaliana* [281]. In contrast, hypermethylated mutants (e.g., *ros1*) alter cell wall defense and SA-dependent gene expression, leading to increased susceptibility to DM [281]. EpiQTL for DM resistance in *A. thaliana* also showed that heritable DNA hypomethylation in pericentromeric regions is associated with the regulation of defense-related genes [282]. In *A. thaliana*, a triple mutant of DNA demethylases (*rdd*: *ros1 dml2 dml3*) shows susceptibility to *Foc* [283]. A reduction in CHH methylation in the *rdd* mutant may participate in DNA demethylase-mediated *Foc* resistance. In contrast, RdDM pathway mutants (*nrpe1* and *ago4*) are susceptible to *Foc*, suggesting that RdDM plays a role in resistance mechanisms [283].

SET DOMAIN GROUP8 (SDG8) is a histone methyltransferase of H3K36me3. Alteration in H3K36me3 levels at *MITOGEN-ACTIVATED* *PROTEIN KINASE 3* (*MKK3*), *MKK5*, and some defense marker genes, caused by *A. brassicicola* infection in the *sdg8-1* mutant confers resistance similar to JA treatment in wild-type *A. thaliana* [284]. Histone H2Bub regulates hyphal growth, conidia formation, and the pathogenicity of *A. alternata* [285]. H2Bub mutants (*hub1*) showed thinner cell walls and changes in surface cutin and wax composition/deposition, resulting in increased susceptibility to fungal pathogens [286,287]. AtHUB1 interacts with AtMED21 to suppress defense response against pathogens [286]. *HISTONE DEACETYLASE 19* (*HDA19*) expression is induced after *A. brassicicola* infection, as well as JA and ET treatments, suggesting that HDA19 plays a role in the resistance mechanisms through the JA-dependent pathway [288]. Knockout of *HDA19* decreases resistance against *A. brassicicola*, whereas its overexpression increases resistance [288]. In *hda19* mutants, the expression level of SA-defense-related genes including *PR1* and *PR2* is upregulated, along with an increase in SA levels. Hyper-acetylation of histone H3 at *PR1* and *PR2* loci was also observed in the *hda19* mutant, suggesting that the activation of *HDA19* is important for the defense response [289]. LIKE HETEROCHROMATIN PROTEIN 1 (LHP1)-Interacting Factor 2 (LIF2) is an RNA-binding family protein and is involved in plant immunity. In *A. thaliana*, the *lif2* mutant exhibits increased resistance against *S. sclerotiorum* by upregulating the SA-mediated defense genes [290]. LHP1 binds to H3K27me3, can interact with LIF2 [291,292]. Thus, the deposition of H3K27me3 marked by LHP1 can repress *LIF2* expression, thereby increasing resistance to *S. sclerotiorum* in *A. thaliana*.

### 4.3. Epigenomic Defense Response in Brassica Vegetables

As introduced in the previous section, the relationship between disease resistance and epigenetic regulation—including the identification of epigenetic QTL (epiQTL)—has been well documented in *A. thaliana*. In contrast, although still limited, emerging studies have begun to uncover similar epigenomic mechanisms in *Brassica* vegetables in response to biotic stress. *Brassica* vegetables undergo pathogen-induced hypo- or hyper-methylation as part of their defense mechanisms [264]. For example, in *A. thaliana*, *Foc* inoculation controls the expression of stress-responsive genes through DNA methylation and demethylation at TE located in promoter regions of genes [283]. In *B. rapa*, DNA methylation in introns and 200 bp up- and downstream regions of genic regions results in transcriptional suppression in both *Foc* susceptible and resistant lines [257]. The results of DNA methylation state that, relative to non-*Foc*-infected samples, 87 and 98 DEGs between *Foc*- and mock-inoculated samples at 24 h after inoculation showed DNA methylation within genic regions in susceptible and resistant lines, respectively, and 36 DEGs were common to both lines [257]. The resistant line had DNA methylation and differential expression for some defense-responsive genes like *JASMONATE-ZIM-DOMAIN PROTEIN 1* (*JAZ1*), *PATHOGENESIS-RELATED 3* (*PR-3*), *WRKY51*, *NON RACE-SPECIFIC DISEASE RESISTANCE 1* (*NDR1*), and *RESPIRATORY BURST OXIDASE HOMOLOGUE D* (*RBOHD*) [257]. *JAZ1* is involved in the jasmonate stimulus. The ethylene/jasmonic acid signaling pathway involves *PR-3*, and ethylene/salicylic acid signaling pathways and jasmonic acid-inducible defense responses are mediated via *WRKY51*. *RBOHD* regulates the production of reactive oxygen intermediates (ROIs) through its interaction with *RESPIRATORY BURST OXIDASE PROTEIN F* (*RBOHF*) to control hypersensitive response (HR) at the pathogen infection site. *NDR1* is essential for non-race-specific resistance to fungal pathogens. It is suggested that DNA methylation regulates transcription of these genes and is crucial for mediating the SAR response against pathogen infection. Differentially methylated regions (DMRs) following *A. candida* inoculation were located within genes in the susceptible variety Misugi, while they were located in upstream and downstream regions of the resistant variety Nanane of *B. rapa* subsp. *perviridis* [293]. DMRs for CG methylation were observed in gene bodies of both Misugi and Nanane. Thirteen DEGs (eight in Misugi and five in Nanane) have a negative correlation between expression levels and DNA methylation levels [293]. Genes encoding NBS-LRR family proteins and genes involved in SA signaling pathways tended to be differently methylated in response to TuMV infection in Chinese cabbage, and alterations of DNA methylation were associated with the activation of the immune response against TuMV [294]. Epigenetic regulation involving histone modifications has also been reported in response to pathogen infection in *Brassica* vegetables. Pathogenesis-related protein encoding gene Bra008226 (*PDF1.2b*, *plant defensin 1.2b*) was densely enriched with H3K27me3 in the *brclf* mutant of *B. rapa*, suggesting the polycomb group proteins mediated epigenetic regulation of biotic stress [295]. Over 20% of genes marked with bivalent histone methylation (H3K4me3 and H3K27me3) before infection tended to show changes in expression following *Foc* inoculation of *B. rapa* [268]. An mRNA and its paired NAT, Bra033549-MSTRG.1355, with H3K4me3 and H3K27me3 marks showed highly coordinated expression following *Foc* inoculation of *B. rapa* [271]. These studies suggest that histone modifications play a role in regulating transcriptional responses during *Foc* infection of *B. rapa*. Despite growing evidence on epigenetic insights, epiRILs are currently unavailable for epigenome mapping and the development of epi-markers in Brassica vegetables. It will be possible to evaluate the stability and effectiveness of epi-markers for resistance breeding in *Brassica* vegetables once epiRILs become available.

## 5. Perspective

**Figure 5:**
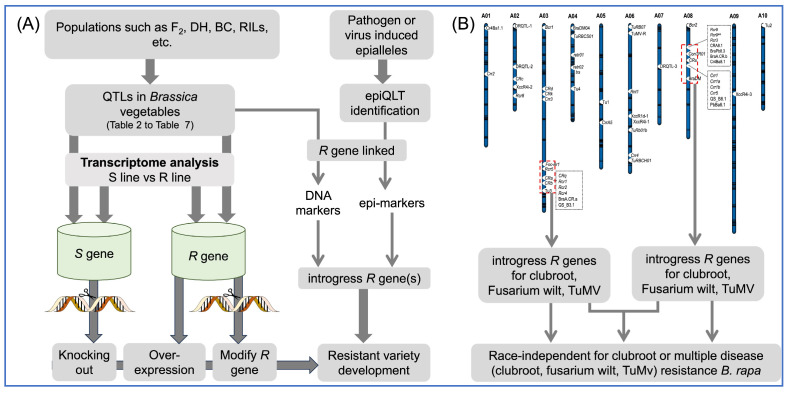
Molecular strategies for the biotic stress resistant variety development (**A**), and introgression of multiple *R* genes in *B. rapa* (**B**). Figure 3 is used to show chromosomal locations. S—susceptible; R—resistant; F_2_—second filial generation; DH—double haploid; BC—backcross; RILs—recombinant inbred lines; QTLs—quantitative trait loci; epiQTLs—epigenetic QTLs; TuMV—turnip mosaic virus.

*Brassica* vegetables face mounting challenges from global climate change. Pathogen infestations have resulted in significant losses in global vegetable production. Integration of genetic and epigenetic insights will aid breeders in developing sustainable resistant breeding strategies for future crop improvement. Current understanding of genomics and transcriptomics in response to biotic stress in *Brassica* vegetables is advancing, while knowledge of the epigenomics remains limited. Researchers have uncovered novel resistance loci, *R* genes, and defense-regulating TFs for key pathogens causing clubroot, Fusarium yellows, downy mildew, black rot, sclerotinia rot, soft rot, Alternaria leaf spot, blackleg, and white rust diseases in *Brassica* vegetables. Introgression of *R* genes is a fundamental strategy for resistant variety development that can be more efficient by the application of molecular markers (Figure 5A). CRISPR-based tools offer unprecedented opportunities for manipulating *R* genes to customize resistance mechanisms. Knocking out susceptible genes resulting in non-functional protein using CRISPR-based tools can interfere with pathogen infection to confer resistance (Figure 5A). CRISPR can also be used to modify genes within resistance QTLs by base editing to generate resistant *Brassica* vegetable varieties. CRISPR-based tools can also be used to boost the natural defense mechanisms of the host plant by engineering the key genes involved in SA and JA pathways. The majority of researchers today concentrate on single-gene resistance, which is inappropriate for future resistance breeding programs because pathogens can rapidly evolve to overcome such resistance. It is possible to develop dual/triple/multiple race- and/or pathogen-resistant varieties by introgressing multiple *R* genes. Multiple disease resistance genes are clustered in *B. rapa*, especially in chromosomes A03 and A08 (Figure 3). Introgression of genes from those hotspots could be a key approach in breeding race-independent types (especially for clubroot) and multiple disease-resistant varieties of *B. rapa* vegetables (Figure 5B). There are fewer disease-resistant QTLs in *B. oleracea* than in *B. rapa*. According to current research outcomes, a disease-resistant hotspot for multiple disease is not clear in *B. oleracea*; however, two genomic loci—the top of the C03 chromosome and the bottom of the C07 chromosome—can be considered for introgressing dual resistance (Figure 4). CRISPR-based tools can assist in precise breeding for multiple-gene resistance. In contrast, multi-omics data will assist breeding decisions by predicting plant–pathogen interactions through machine learning and artificial intelligence. These models can translate genotype to phenotype and identify complex resistance mechanisms.

Epigenomic studies in *Brassica* vegetables and *A. thaliana* suggest the importance of epigenetic regulation in controlling plant immune responses. In *A. thaliana*, growing evidence points to epigenetic defense mechanisms against pathogens that could be applied to *Brassica* vegetables. In both *A. thaliana* and *Brassica* vegetables, DNA methylation or histone modifications regulate gene expression under biotic stresses and can mediate rapid and reversible responses to pathogen attack. These rapid and reversible dynamics of gene expression against pathogens will facilitate the breeding of quick defense-activating and long-term adaptation of resistant varieties of *Brassica* vegetables, extending beyond traditional genetic methods. Transgenerational inheritance of epigenetic states can also include prime enhanced resistance in future generations. Understanding the roles of SAR and hormonal regulations mediated by the DNA methylation or histone modifications will help in developing novel resistance breeding strategies in *Brassica* vegetables. Current single-cell epigenomic and transcriptomic technologies will further advance our understanding of tissue-specific resistant mechanisms, aiding the development stage-specific defense systems in resistant varieties. In plants, epialleles with DMRs are often generated under stress conditions, leading to variability in disease resistance. Typically, pathogen/microbial infection increases genome-wide DNA methylation levels with increasing expression of many genes and reducing resistance in host plants. In contrast, a decrease in DNA methylation in pathogen-infected host plants could confer long-term resistance through the evolution of novel *R* genes. The application of demethylating agents (e.g., 5-azacytidine) can partially reduce DNA methylation levels [296,297]. Epigenomic insights will lead to epigenome editing and development of epialleles in *Brassica* vegetables, extending beyond mechanical modifications of epigenomic states. The development of epiRILs in *Brassica* vegetables and identification of epiQTL linked to *R* genes, along with their associated epi-markers, would aid breeders in developing more effective and long-lasting disease-resistant varieties (Figure 5). Validation of newly developed resistant varieties under field conditions will be crucial. Although epigenome editing has enormous potential for resistance breeding, there are several obstacles to overcome. The most significant challenges, regardless of plant species, are off-target epigenetic alteration [297] which could lead to an unexpected phenotype. Finally, the combination of genetic and epigenetic knowledge will boost the development of next-generation resistant *Brassica* varieties, not only for addressing current threats but also for adapting to future environments in safeguarding nutrition and productivity in a rapidly changing world.

## Figures and Tables

**Figure 1:**
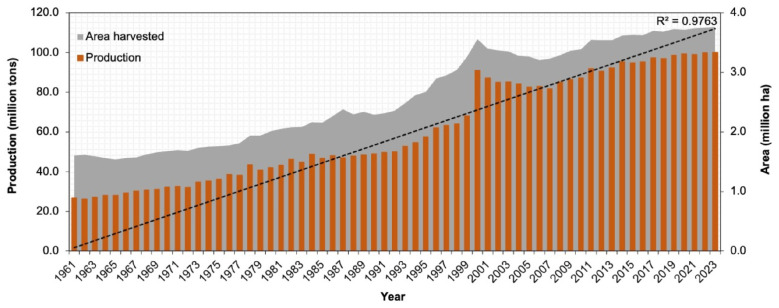
Global production and area of cultivation of *Brassica* vegetables (excluding turnip) by year (data from FAO 2023 [1]). A linear regression line indicates the trend of global production of *Brassica* vegetables.

## Data Availability

Not applicable.

## Abbreviations

4MI3G	4-methoxyindole-3-ylmethyl glucosinolate
4OH-I3M	4-methoxy indole-3-ylmethyl glucosinolate or 4-methoxyglucobrassicin
5mC	5-methylcytosine
ac	Acetylation
*ACA1*	*ALPHA CARBONIC ANHYDRASE* 1
ACCox1	1-amino-cyclopropane-1-carboxylic acid oxidase
AFLP	Amplified fragment length polymorphisms
AS	Alternative splicing
BAC	Bacterial artificial chromosome
BC_1_	Backcrossed first generation
BC_1_F_1_	First filial generation of the first backcross
BC_1_F_2_	Second filial generation of the first backcross (comes from selfing of BC_1_F_1_)
BC_1_S_1_	Backcross segregating first generation
BC_2_	Backcrossed second generation
BC_3_	Backcrossed third generation
BC_4_F_2_	Second filial generation of the fourth backcross
bp	Base pair
BSA-Seq	Bulked segregant analysis sequencing
BSR-seq	Bulked segregant RNA sequencing
*CALM*	*CALMODULIN*
CanF	Canadian field isolates
CAPS	Cleaved amplified polymorphic sequences
CC	Coiled-coil
*CKX*	*Cytokinin dehydrogenase/oxidase*
ConTR01	Conditional TuMV resistance 01
CR	Clubroot resistance
CYP81F2	Cytochrome P450, family 81, subfamily F, polypeptide 2
DAMP	Damage-associated molecular pattern
dCAPS	Derived cleaved amplified polymorphic sequences
DH	Double haploid
DM	Downy mildew
DMG	Differentially methylated gene
DMR	Differentially methylated region
DUS	Distinctness, uniformity, and stability
EFR	Elongation factor-Tu receptor
epiQTL	Epigenetic quantitative trait locus
epiQTLs	Epigenetic quantitative trait loci
ET	Ethylene
ETD	Effector-triggered defense
ETI	Effector-triggered immunity
F_1_	First filial generations
F_2_	Second filial generations
F_3_	Third filial generations
F_4_	Fourth filial generations
FLS	Flagellin-sensing receptor
*Foc*	*Fusarium oxysporum* f.sp. *conglutinans*
*For*	*Fusarium oxysporum* f.sp. *rapae*
FY	Fusarium wilt/yellows
GBS	Genotyping-by-sequencing
GRAS-Di	Genotyping by random amplicon sequencing–direct
H2Bub1	Monoubiquitination of H2B
H3ac	Acetylation of histone H3
H3K27me3	Trimethylation of histone H3 at lysine 27
H3K36me3	Trimethylation of histone H3 at lysine 36
H3K4me3	Trimethylation of histone H3 at lysine 4
H3K9me2	Dimethylation of histone H3 at lysine 9
H4ac	Acetylation of histone H4
*HDA19*	*HISTONE DEACETYLASE 19*
*Hp*	*Hyaloperonospora parasitica*
hpi	Hours post-inoculation
HR	Hypersensitive responses
I3G	Indole-3-ylmethyl glucosinolate
IBP	Intron-based polymorphic
IGMT1	Indole glucosinolate methyltransferase 1
IGMT2	Indole glucosinolate methyltransferase 2
incRNAs	Intronic non-coding RNAs
InDel	Insertion–deletion
IRRs	Interspersed repeat regions
ISSR	Inter-simple sequence repeat
JA	Jasmonic acid
*JAZ1*	*JASMONATE-ZIM-DOMAIN PROTEIN 1*
KASP	Kompetitive Allele-Specific PCR
Kb	Kilobase
Km	Kamogawa
LF	Less fractioned
LG	Linkage group
LHP1	LIKE HETEROCHROMATIN PROTEIN 1
LIF2	LHP1-Interacting Factor 2
lincRNAs	Long intergenic non-coding RNAs
LRR	Leucine-rich repeat
MAMP	Microbe-associated molecular patterns
MAPK	Mitogen-activated protein kinase
MAS	Marker-assisted selection
Mb	Mega base
me	Methylation
MF	More fractioned
MIP	MITE insertion polymorphism
*MKK3*	*MITOGEN-ACTIVATED PROTEIN KINASE 3*
NAT	Natural antisense transcript
NB	Nucleotide-binding
NBS	nucleotide-binding site
*NDR1*	*NON RACE-SPECIFIC DISEASE RESISTANCE 1*
NGS	Next-generation sequencing
NLRs	NB-LRR receptors
*PAL*	*PHENYLALANINE AMMONIALYASE*
PAMPs	Pathogens/microbes via pathogen-associated molecular patterns
*Pb*	*Plasmodiophora brassicae*
*Pcc*	*Pectobacterium carotovorum* subsp. *carotovorum*
PCR	Polymerase chain reaction
*PDF1.2b*	*plant defensin 1.2b*
PGIP	A polygalacturonase inhibitor protein
ph	Phosphorylation
*PR*	Pathogenesis-related
PR	Physiological race
*PR-3*	*PATHOGENESIS-RELATED 3*
PRR	Pattern-recognition receptor
PTI	Pattern-triggered immunity
PTM	Post-transcriptional modification
*PUB1*	Leaf pubescence loci
QTL	Quantitative trait locus
QTLs	Quantitative trait loci
*R*	Resistance
R	Resistant line
RAD-seq	Restriction-site Associated DNA Sequencing
RAPD	Random amplified polymorphic DNA
*RBOHD*	*RESPIRATORY BURST OXIDASE HOMOLOGUE D*
*RBOHF*	*RESPIRATORY BURST OXIDASE PROTEIN F*
RH	Relative humidity
RIL	Recombinant inbred line
RLKs	Receptor-like kinase
RLP	Receptor-like protein
RNAi	RNA interference
ROI	Reactive oxygen intermediate
*RPS2*	*RESISTANT TO P. SYRINGAE 2*
*RPS4*	*RESISTANT TO P. SYRINGAE 4*
RRBS	Reduced representation bisulfite sequencing
S	Susceptible line
SA	Salicylic acid
SAR	Systemic acquired resistance
SCAR	Sequence-characterized amplified region
SHPRH	SNF2—sucrose non-fermenting2, histone linker, PHD—plant homeodomain, RING—really interesting new gene, helicase
SLAF	Specific-locus amplified fragment
SNP	Single-nucleotide polymorphism
SR	Sclerotinia rot or stalk rot
SRAP	Sequence-related amplified polymorphism
SSR	Simple sequence repeats
STS	Sequence-tagged site
TDF	Transcript-derived fragment
TE	Transposable element
TF	Transcription factor
TIR	N-terminal Toll/Interleukin-1 receptor
TIR	Toll/Interleukin-1 Receptor
TuMV	Turnip mosaic virus
ub	Ubiquitination
UGMS	Unigene-derived reliable microsatellite
W01	Wakayama-01
WAK	Wall-associated kinase
WGBS	Whole genome bisulfite sequencing
*Xcc*	*Xanthomonas campestris* var. *campestris*
YCR	Fusarium yellows and clubroot resistance
YR	Fusarium yellows resistance
